# Review on Natural, Incidental, Bioinspired, and Engineered Nanomaterials: History, Definitions, Classifications, Synthesis, Properties, Market, Toxicities, Risks, and Regulations

**DOI:** 10.3390/nano12020177

**Published:** 2022-01-06

**Authors:** Ahmed Barhoum, María Luisa García-Betancourt, Jaison Jeevanandam, Eman A. Hussien, Sara A. Mekkawy, Menna Mostafa, Mohamed M. Omran, Mohga S. Abdalla, Mikhael Bechelany

**Affiliations:** 1 NanoStruc Research Group, Chemistry Department, Faculty of Science, Helwan University, Helwan 11795, Egypt; Eman2022@science.helwan.edu.eg (E.A.H.); mamahmoud@science.helwan.edu.eg (M.M.); 2 School of Chemical Sciences, Dublin City University, D09 V209 Dublin, Ireland; 3 Centro de Investigaciones Químicas, IICBA, Universidad Autónoma del Estado de Morelos, Cuernavaca 62209, Mexico; mluisa.garcia@uaem.mx; 4 CQM—Centro de Química da Madeira, MMRG, Campus da Penteada, Universidade da Madeira, 9020-105 Funchal, Portugal; jaison.jeevanandam@staff.uma.pt; 5 Chemistry Department, Faculty of Science, Helwan University, Helwan 11795, Egypt; saraahmed9572_892@science.helwan.edu.eg (S.A.M.); drmmomran@science.helwan.edu.eg (M.M.O.); mohga.shafiek@science.helwan.edu.eg (M.S.A.); 6 Institut Européen des Membranes, IEM, UMR 5635, Université Montpellier, ENSCM, CNRS, 34000 Montpellier, France

**Keywords:** nanoparticles, nanostructures, nanosystems, synthesis methods, size-dependent properties, shape-dependent properties

## Abstract

Nanomaterials are becoming important materials in several fields and industries thanks to their very reduced size and shape-related features. Scientists think that nanoparticles and nanostructured materials originated during the Big Bang process from meteorites leading to the formation of the universe and Earth. Since 1990, the term nanotechnology became very popular due to advances in imaging technologies that paved the way to specific industrial applications. Currently, nanoparticles and nanostructured materials are synthesized on a large scale and are indispensable for many industries. This fact fosters and supports research in biochemistry, biophysics, and biochemical engineering applications. Recently, nanotechnology has been combined with other sciences to fabricate new forms of nanomaterials that could be used, for instance, for diagnostic tools, drug delivery systems, energy generation/storage, environmental remediation as well as agriculture and food processing. In contrast with traditional materials, specific features can be integrated into nanoparticles, nanostructures, and nanosystems by simply modifying their scale, shape, and composition. This article first summarizes the history of nanomaterials and nanotechnology. Followed by the progress that led to improved synthesis processes to produce different nanoparticles and nanostructures characterized by specific features. The content finally presents various origins and sources of nanomaterials, synthesis strategies, their toxicity, risks, regulations, and self-aggregation.

## 1. Introduction

Nanotechnology combines distinct domains (engineering, chemistry, physics, biology, and medicine) with the ultimate aim of creating ‘things’ at the scale of atoms. One nanometer (i.e., one billionth of a meter) is the diameter of a hydrogen atom. The term nanotechnology (or ‘“nanotech”) used in recent times describes the manipulation of matter. The manipulation by chemical, molecular, and supramolecular methods fabricate nanoparticles (NPs), nanomaterials, and nanostructured materials. Nanotechnology interests also in studying the specific features observed upon modification (particularly reduction) of the size of the nanoparticles [1]. In particular, tiny nanoparticles with sizes <10 nm exhibit exclusive properties compared with bulk particles. Moreover, size reduction dramatically alters the nanomaterial’s optical, electrical, and magnetic features. For example, 20 nm diameter Au NPs have a localized surface Plasmon resonance at a wavelength of 520 nm in water, which red shifts to 600 nm as the Au NPs diameter increases to 100 nm [1].

Nowadays, nanotechnology is rapidly expanding with new applications in different fields. For instance, nano-engineering brings new opportunities for progress in healthcare, energy, environmental protection, constructions, agriculture food processing, and other fields by developing nanomaterials, nanostructures, and nanosystems. To date, different research fields, from organic chemistry to semiconductor physics, are implicated in the fabrication of many different nanoparticles and nanostructured materials and also in the related applications, from designing new nanomaterials to the direct atomic-scale regulation of matter [1]. Researchers are currently working to develop nano-robots that can choose, pick, and place atoms, in the same way as children use LEGO bricks. According to Global Industry Analysts, Inc., nanotechnology size was estimated at US$42.2 billion in 2020, and should increase to $70.7 billion by 2026.

Nanomaterials differ in their dimensions, shapes, sizes, compositions, porosity, phases, and uniformity and thus, several classifications have been used to categorize them. To date, many types of nanomaterials have been described and many more will be developed. Nanomaterials can also be classified into naturally occurring, incidental, bioinspired, and engineered nanomaterials in function of their origin [2]. Naturally occurring nanomaterials form during natural physicochemical processes. Incidental nanomaterials, also known as anthropogenic or waste particles, occur because of man-made industrial processes [3]. Engineered nanomaterials are fabricated in the laboratory/industry to obtain materials with specific features. Bioinspired are engineered nanomaterials the properties of which mimic those of natural nanomaterials or living matter [4]. Engineered and bioinspired nanomaterials are gaining attention among researchers, but they represent only a small part (in terms of mass) relative to the natural nanomaterials’ varieties [5,6,7]. Despite their exclusive properties, few engineered and bioinspired nanomaterials have been approved and used in the industry, due to the need for large-scale production, and risk assessment processes.

This review provides an overview of nanoparticles and nanostructured materials, including their history, definitions, classifications, unique properties, toxicity, risks, and regulations. It presents a systematic evaluation of prehistoric nanomaterials, historical evidence of nanomaterials utilization in ancient civilizations (4000 BC in Egypt, Indonesia, China), Roman Empire and medieval period, and modern definitions of nanoscience and nanotechnology development and the underlying science. Moreover, it summarizes definitions, marketing and international regulations on nanomaterials. Different classifications of nanomaterials based on their size, shape, origin, compositions, and other specific features are presented in a systematic manner. Various fabrication techniques to produce nanomaterials, under different names and classifications, are covered in detail. The review also discusses the nanomaterial exposure routes in the human body, the organs/tissues concerned, and the diseases linked to such exposure (based on published epidemiological, in vivo, and in vitro data).

## 2. History and Development of Nanomaterial Research

**Figure 1:**
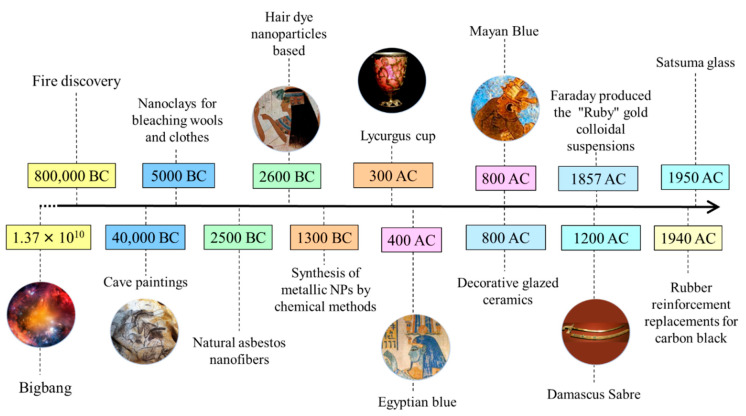
Timeline summarizing ancient civilizations; they produced and used nanomaterials without knowing their nanosized characteristics.

Forest fire products, volcanic ash, ocean spray, radioactive decay, and weathering processes of metal- or anion-containing rocks are among the natural sources of nanomaterials. It is thought that nanoparticles and nanostructured materials originated from meteorites generated during the Big Bang process that led to the formation of the universe and Earth. Nanodiamonds extracted from meteorites are considered the most abundant type of presolar grain [8]. Naturally occurring nanomaterials have been intimately associated with life development on the Earth, from the first cells to humans. Nanomaterials and their derivatives have been produced and employed by humans since prehistoric times. Due to major advances in nanomaterials characterization techniques [9,10], it is becoming possible to better assess all nanomaterial types and their origins can be studied of nanomaterials in Earth systems and to better frame their longer-term effects impact on the Earth environmental and on human health can be estimated. Figure 1 shows the timeline of nanotechnology developments in various ancient civilizations and distinct epochs (prehistoric, ancient, medieval, modern, and contemporary). The contemporary period in nanomaterials development lasted until 1959, and modern nanotechnology was initiated in the 1960s.

### 2.1. Prehistoric Nanomaterials

**Figure 2:**
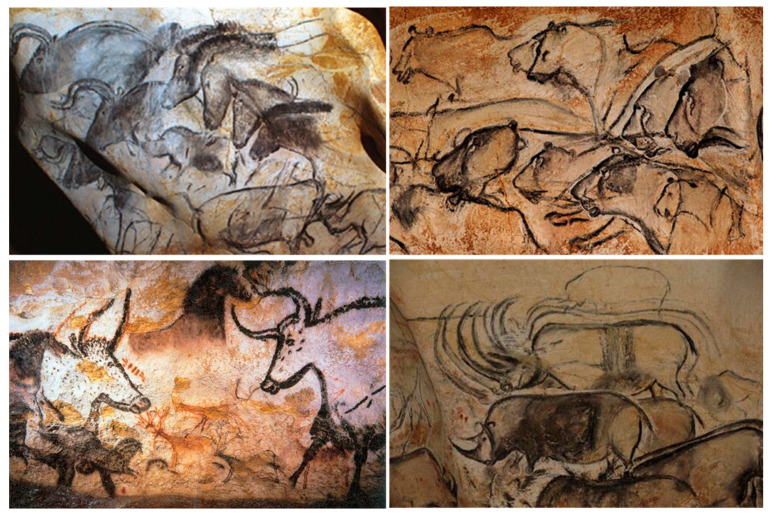
Ancient Paintings in Chauvet-Pont-d’Arc and Lascaux caves containing nanostructures. © MCC/DRAC. Open Access.

Since prehistoric times, humans have been using fire. The smoke and soot of such fires contained nanoparticles (e.g., fullerenes, graphene, and carbon nanotubes) and other combustion by-products. Our ancestors used these carbon nanomaterials and waxes for cave paintings. Hand stencils in caves of Sulawesi, Indonesia, are the oldest known examples of art expression by humans. The carbon dating results showed that these cave paintings were created in 40,000 BCE using fat, charcoal, and plant pigments [11,12,13]. Similar cave paintings are visible also in the Chauvet-Pont-d’Arc cave, France (date at 34,000 BCE). Analysis of cave paintings suggests that people of ancient civilization used nanomaterials, such as graphene, without knowing it (Figure 2) [13].

### 2.2. Nanomaterials and Ancient Civilizations

**Figure 3:**
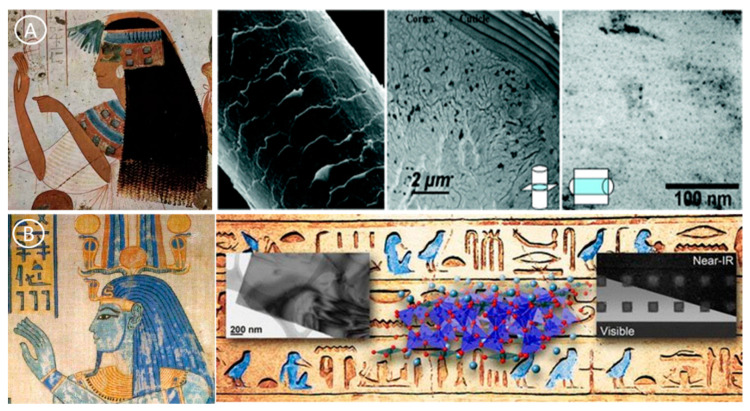
Nanomaterials produced by humans in ancient civilizations: (**A**) Egyptians produced PdS_2_ NPs as a dye for hair [16], © American Chemical Society, 2006; (**B**) Egyptians produced Egyptian blue (CaCuSi_4_O_10_ and SiO_2,_ nanosheets of less than 5 nm in thickness) [17], © American Chemical Society, 2013.

While nanomaterials appear as a new term at the end of the 19th century, nanomaterials have been produced, and used already in ancient civilizations. In ancient Egypt, the soot of oil lamps was used to make black pigments of high opacity and stability for writing on papyrus. At that time, Egyptians did not know that soot contains carbon nanomaterials. Clay minerals, nano-platelets about 1 nm in thickness, are among the best examples of natural nanomaterials used in ancient times (5000 BC). Humans manipulated ceramic matrix materials reinforced with natural asbestos nanofibers (50–200 nm in diameters) earlier than 4,500 years ago [14]. For instance, they were used for wool and cloth bleaching in Cyprus [15]. Ancient Egyptians used synthetic chemical processes for producing the fabrication of PdS_2_ NPs (with a diameter of ~5 nm in diameter), as ingredients for the preparation of hair colorants (Figure 3A) [16].

Sometime around 2575 BC, Egyptians were already producing a synthetic pigment (Egyptian blue, nanosheets of 5 nm in thickness), based on a sintered mixture of nanosized glass and quartz (CaCuSi_4_O_10_ and SiO_2_) (Figure 3B) [17]. Similarly, a blue pigment phase was produced in China in which Ba (Ba Cu Si_4_O_10_) replaced the Ca used in Egypt. Egyptian blue was massively used for decorative purposes in many countries that were part of the Roman Empire [17]. In the Americas, the ancient Mayas civilization produced synthetic pigment composites (including blue) in the 8th century BC. These dyes have survived and resisted humidity in pottery, murals, and ceremonial artifacts. Interestingly, the material composition of Egyptian blue is different from that of the blue pigments produced by other ancient civilizations in Europe and Asia. Several studies suggested that the bright color of Egyptian blue might be explained by the presence of an inorganic pigment with nanostructured clays and a low quantity of organic components (*Indigofera suffruticosa* leaves in water) [18,19,20].

The first metallic nanoparticles were produced by Egyptian and Mesopotamian glass manufacturers using chemical methods already in the 14th and 13th centuries BC [21]. Red glass, due to the excitation of surface plasmons in copper (Cu) nanoparticles, prepared in 1200–1000 BC (late bronze age) was discovered in Italy [22]. Similarly, Celtic red enamels produced between from 400 to 100 BC contain Cu NPs and cuprous oxide (cuprite Cu2O) [23]. Mayan Blue, an azure pigment that is resistant to corrosion and discovered in the pre-Columbian Mayan city of Chichen Itza, was first fabricated in 800 AD. Maya Blue is composite nanomaterials in which indigo dye was combined chemically with clay nanopores to obtain an environmentally stable dye [24]. Cementite nanowires and carbon nanotubes were identified in the microstructure of wootz steel produced in India in 900 AD [25]. In Cyprus, clay was already used in 5000 BCE for cloth bleaching [21].

### 2.3. Nanomaterials in Medieval Times

**Figure 4:**
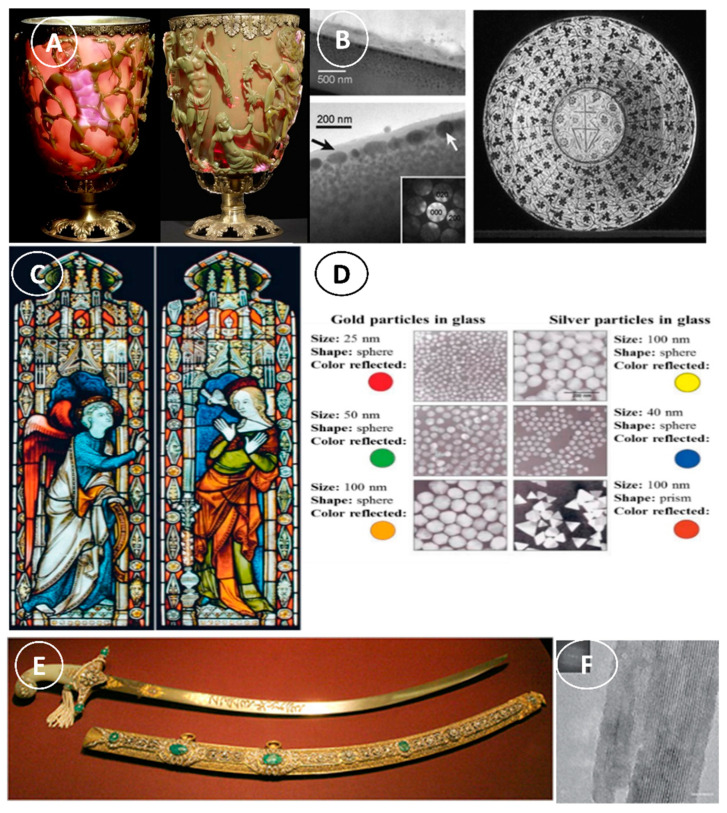
Examples for some handmade nanomaterials used in medieval times. (**A**) The Lycurgus cup is brilliant red when light passes through the glass that contains Au-Ag NP alloy (The Trustees of the British Museum/Art Resource, NY). (**B**) Ag NPs in ancient ceramic plates [35]. © Wiley, 2004 (**C**) Nanoparticles in medieval church windows [36]. © MDPI, 2000 (**D**) Transmission electron microscopy (TEM) images of the Au and Ag NPs used in colored window glasses in medieval churches. (**E**) Saber made of Damascus steel (photograph by Tina Fineberg for The New York Times); (**F**) TEM image of CNTs in a Damascus Saber after dissolution in hydrochloric acid, showing some cementite nanowires encapsulated by carbon nanotubes (scale bar, 5 nm) [34]. © Nature, 2006.

The common utilization of NPs (particularly metallic NPs) and nanostructures by Romans is one of the most significant illustrations of ancient nanomaterials usage and nanotechnology knowledge in ancient times. One of the most impressive examples illustrations of the Roman glass manufacturing is the Lycurgus cup (Figure 4A), the oldest known dichroic glass that displays two distinct colors when exposed to different light wavelengths. The Lycurgus cup looks green under direct light, and red-purple when lit from the back (Figure 4A) [26]. Transmission electron microscopy analysis showed that the dichroism is caused by the presence of nanoparticles of 50–100 nm in diameter in the glass [27]. X-ray analysis (XRD) allowed identifying some of these nanoparticles in the glass matrix, particularly silver-gold (Ag-Au NPs) alloys (Ag:Au ratio of ~7:3) and ~10% of Cu NPs [28,29]. The Au NPs emit a red color (~520 nm) due to light absorption. The red-purple and green colors are explained by the light absorption by larger Au particles and by the light dispersion in colloidal dispersions of Ag NPs bigger than 40 nm, respectively.

For many centuries (9th to17th), lusterware was first very popular in Islamic countries and then also in Europe. Elemental analysis of these ceramic glazes showed the presence of Ag, Cu, and other NPs [30]. Transmission electron microscopy analysis revealed two thin layers of Ag NPs (5–10 nm) in the outer surface and thicker layers (5–20 nm) in the inner part (Figure 4B). The two layers are separated by ~430 nm, and this explains their optical interference property. The light reflected by the second-thick layer contributes to the phase change, depending on the light scattering by the first layer. This phase-dependent alteration of the incoming light wavelength leads to a different scattering process in function of the wavelength. Later, this technique was exploited to fabricate red glass worldwide. In the mid-19th century, Satsuma glass was produced in Japan using a similar method. The inclusion of Cu NPs in the glass explains the bright ruby-color of Satsuma glass [31].

Similarly, in the glass windows of late medieval churches, the bright red and yellow colors are the results of the inclusion of Au and Ag NPs in the glass in its molten state (Figure 4C,D) [32]. The early alchemists were also fascinated by gold. They tried to produce potable gold known as ‘‘the elixir of life’’, because they considered gold as indestructible and with enormous medicinal importance [32]. During the Renaissance Period (1450–1600AD), the people in Umbria (Italy) used nanotechnology to produce iridescent or metallic glazes. They achieved these effects by using Cu and Ag NPs (5–100 nm) in their glazes, this caused light to bounce off their surface at different wavelengths, thus giving it the ‘iridescent’ look [33]. Moreover, in the 13th–18th centuries, “Damascus” steel was used to manufacture blades. This steel contained cementite nanowire, and carbon nanotubes (CNT) to give power, toughness, and sharpness (Figure 4E,F) [34]. Noteworthy, nanomaterials could be identified in all these ancient materials after the development of imaging facilities to characterize the nanomaterials morphology.

### 2.4. Nanomaterials in the Modern Age

**Figure 5:**
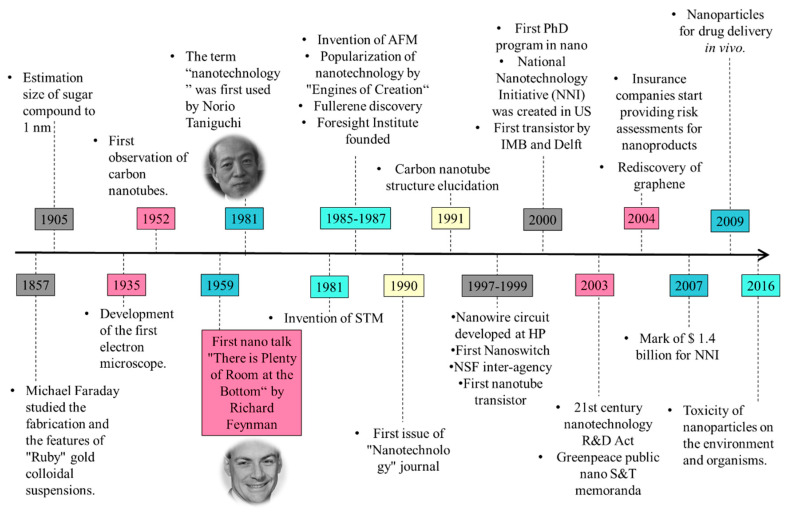
Timeline of nanomaterials discovery in the modern nanotechnology era.

The modern age of nanotechnology and the story of this magnificent discovery takes us back to a man named Michael Faraday (Figure 5) who investigated the properties of light and matter in the mid-1850s. In 1857, he studied the fabrication and the features of “Ruby” gold colloidal suspensions and showed that Au NPs can alter the solution color under certain light conditions [37]. In 1905, Albert Einstein estimated the diameter of a sugar molecule as ~1 nm. In the 1940s, SiO_2_ NPs were fabricated as rubber reinforcement replacements for carbon black [38]. The development of electron microscopy was the breakthrough in modern nanotechnology. In 1935, Ernest Ruska and Max Knoll constructed the first electron microscope [39]. Conventionally, different electron sources made of tungsten, including tipped semiconductor field emitters, were used to improve the visualization of materials and nanomaterials. The application of field emission via nanostructured semiconductors led to the emergence of better-resolution scanning electron microscopes [40]. Another important and revolutionary contribution to nanotechnology was the development of the scanning tunneling microscope by Gerd Binning and Heinrich Rohrer to acquire high-resolution images of nanomaterials [39,41,42]. In resume, Figure 5 presents the most remarkable events and discoveries that defined modern nanotechnology.

In 1952, Radushkevich and Lukyanovich were the first to describe carbon nanotubes [43] by electron microscopy. The lecture entitled “There is plenty of room at the bottom” by Richard. P. Feynman at the American Physical Society yearly meeting on 29 December 1959, opened a whole new field, known as ‘nanotechnology’ [1]. In the early 1960s, only some researchers, such as Richard Zsigmondy, foresaw the future of nanoscience (“nano-future”) and introduced the term “nanometer” to refer to the measurement of gold colloidal dimensions [44]. At a conference in 1974, Norio Taniguchi proposed to use “Nanotechnology” to describe the manipulation of nanomaterials [39,41,42].

In 1986, the book “Engines of Creation: The Coming Era of Nanotechnology” was published by Erick Drexler to popularize the idea of nanotechnology. In this work, nanotechnology is presented as a revolutionary field [45]. Concomitantly, carbon allotropes gained attention among researchers. However, the “boom” of nanotechnology and the interest in carbonaceous materials increased particularly after the elucidation of the carbon nanotube structure by Sumio Ijima [46]. In 1996, F. Curl, Harold W. Kroto, and Richard E. Smalley were awarded the Nobel prize for the discovery of fullerenes in 1985. Moreover, in 2010, Andre Geim and Konstantin Novoselov received the Nobel Prize in Physics for the physical properties at room temperature of graphene delaminated from graphite [47,48].

Recently, many nanomaterials have been developed dramatically enhanced the features of bulk materials such as strength, conductivity, toughness, lightness, to add new interesting characteristics such as self-healing, self-cleaning, anti-freezing, anti-bacterial, and so forth [49]. Recently, they are used as building material reinforcement or safety sensing components or for other biomedical applications, e.g., biosensors and drug delivery systems [50,51]. In addition to all these potential advantages, nanoparticles are widely used simply because of their size- and shape-specific effects that enhance the material outward aspect. In addition, nanomaterials used in the industry mostly concern nanomaterials embedded in a nanocomposite, resulting in the formation of inert (polymer or cement) or matrix materials [52].

### 2.5. Contemporary Nanomaterials in Material Engineering Applications

The global nanomaterials market size was estimated at USD 8.0 billion in 2020 and is expected to reach USD 9.4 billion in 2021. It also is expected to expand at a compound annual growth rate (CAGR) of 14.1% from 2021 to 2028. Excellent physio-chemical properties and growing usage of nanomaterials in electronics, healthcare, aerospace, and textiles industries are expected to drive the market over the forecast period. The use of engineered nanofibers already makes clothes water- and stain-repellent or wrinkle-free [53,54]. A sunscreen based TiO_2_ nanoparticles offer a comparable UV-protection property. Shoes soccer, football, and baseball already on the market are made with CNTs that reinforce the resin, which is said to improve its performance by making it lighter. Carbon, metals and metal oxide nanomaterials have been used to improve the military’s ability to detect biological agents. By using nanotechnology, the military would be able to create sensor systems that could detect biological agents and drugs with high sensitivity and low detection limits [55,56,57,58,59]. In the polymer manufacturing industry, nanoparticles and nanostructured materials are exploited as reinforced materials [60], e.g., and tire fillers (to increase road adhesion), in the car body (to increase rigidity), and as transparent layers in heat-insulating, moisture-free, and frost-free car windows [61]. In 2003, Mercedes-Benz introduced nanoparticles-based clear metallic and non-metallic coating paints to improve the car resistance to scratch and increase gloss. Liquid magnets (also known as ferrofluids) are ultra-stable magnetic nanoparticles suspensions that display super-paramagnetic features [62]. When an external magnetic field is applied, the nanoparticles in the suspension become aligned in the magnetic field direction [63]. Commercial solar cells fabricated using TiO_2_ NPs exhibited dye-sensitization capacities [64]. In 2012, Logitech marketed an iPad keyboard powered by solar irradiation, using dye-sensitized solar cells. Abraxane^TM^ sold bound nanoparticles form of paclitaxel bound to human albumin in 2005; FDA approved such nanoparticles for cancer treatment [65]. In 2014, more than 1800 nanotechnology-based consumer products were commercialized in more than 20 countries [66].

## 3. Nanomaterial Market Size

The nanomaterial market size should substantially expand during the 2021–2027 period because nanotechnology is progressively more implemented in many industrial sectors [4]. Nanotechnology plays an important role in the aerospace and defense industries and also in other economic sectors, from healthcare and cosmetics to food/beverages, home and garden, as well as electronics and computational analytics [67]. Moreover, green synthesis methods are progressively implemented in order to produce nanomaterials in a non-toxic and eco-friendly manner [68]. The global market of nanomaterials was estimated at US$7.1 billion in 2020 (during the COVID-19 epidemics), and should reach US$12.1 billion by 2026, with a compound annual growth rate of 9.7% [69]. Altair Nanotechnologies, EMFUTUR Technologies, Southern Clay Products, Fuso Chemicals, DuPont, Evonik Industries (RAG-Stiftung), Bayer, BASF, and Ahlstrom-Munksjö, are few of the main players in the global nanomaterials industry. According to Global Industry Analysts, Inc., the nanomaterial market in the United States (US) was US$2.1 billion 2021, and in China, it should reach US$1.2 billion by 2026 (compound annual growth rate of 11.4% for the studied period). Similarly, in Japan, Canada and Germany, the compound annual growth rate is estimated at 8.1%, 8.7%, and 9.1%, respectively, for the same period, The US National Nanotechnology Initiative reported a progressive increase in federal government grants for nanotechnology projects from $464 million in 2001 to ~$6.2 billion in 2019. Similarly, the nanotechnology investments by the European Union (EU) and Japan [70,71]. Similarly, the nanotechnology investments by the European Union (EU) and Japan progressed from ~$1.5 and $1.8 billion, respectively, in the 2005–2010 period to ~$3–4 billion in the 2019–2020 period [72,73,74]. In the 2010–2020 period, $300, $250, and $110 million were allocated to nanotechnology projects by South Korea, China, and Taiwan, respectively [75,76]. The report “The Global Nanomaterials Market, 2010–2025” [77] thoroughly analyzed the current nanomaterials market and its projected developments [78]. Nanomaterial emergence has drastically changed the passenger car sectors (costs, fuel efficiency, and size). For instance, in 2009, Tata Motors (India) put on the market Tata Nano, the cheapest car in the world (INR 100,000). They made it possible because nanomaterials in the components increased the engine efficiency and reduced the total weight of the car [79]. Moreover, carbon black nanoparticles from 10 to 500 nm in size are now used as fillers in the car tire polymer matrix and to increase the mechanical strength [4].

## 4. Nano Terminologies and Standard Definitions

**Figure 6:**
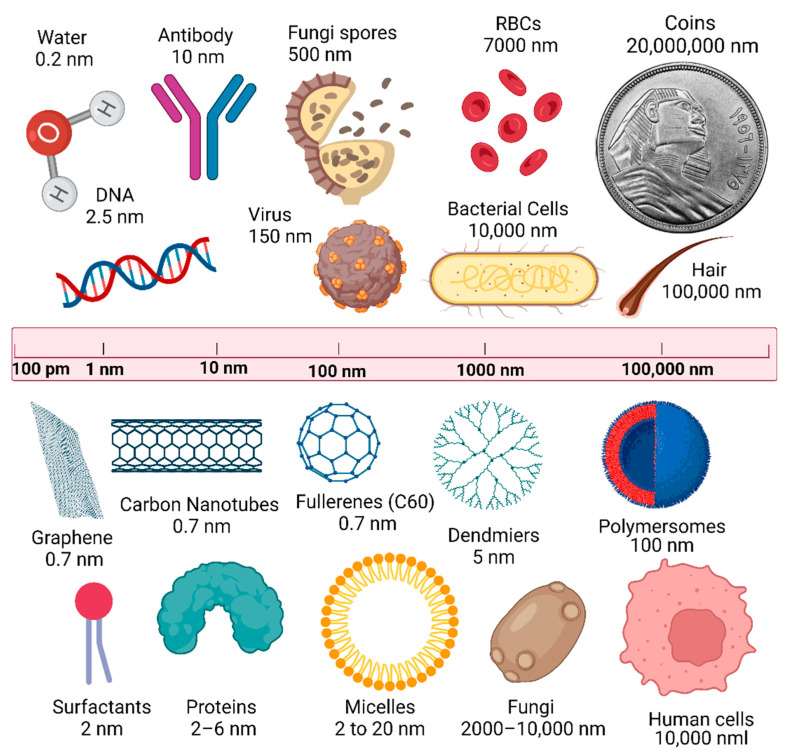
Scheme showing the size scale of objects compared with the nanoscale size regime.

The prefix ‘nano’ derives from the Greek word ‘nanos’ (i.e., ‘very small’), which describes the nanoscale (1–100 nm) considered suitable for particles and materials [36]. Generally, any material classified as a nanomaterial has a size between 1–100 nm, and its properties should be different/enhanced compared with the bulk counterparts [80]. In comparison, a single human hair is between 60,000 and 100,000 nm in thickness, a red blood cell is ~7000 nm in diameter, paper is ~75,000 nm thick, and the DNA double helix radius is 1 nm, and the radius of isolated neutral atoms is between 30 and 300 pm (trillionths of a meter) (Figure 6). Therefore, the radius of an atom is 10,000 times bigger than the radius of its nucleus (1–10 fm), and less than 1/1000 of the visible light wavelength of (400–700 nm).

Nanotechnology and nanoscience are two research areas that focus on matter at nanometer scale. If any research involves with objects less than 100 nm, it belongs to one of nanotechnology and nanoscience. Nanotechnology Standards Panel, established by the American National Standards, released the following nanotechnology standardization priorities: (i) to define the terminologies to describe the structure and features of materials that emerge from nanoscience and nanotechnology [81], (ii) to develop metrology research and standard test methods [82], and (iii) to evaluate the toxicity, environmental impacts, and risk assessment of these new materials [83]. In contrast with nanotechnology, nanoscience describes the study of matter at the nanometer scale, especially the size-dependent characteristics properties of solid-state materials. Researchers started to be interested in this topic since the first studies on the sizes of atoms. Nanoscience combines different disciplines (physics, materials science, and biology) to understand and manipulate materials at the atomic and molecular scales. On the other hand, nanotechnology describes the capacity to observe, measure, manipulate, and produce manufacture nano-matter.

To date, several agencies and regulatory committees have provided scientific terms. (e.g., nanoparticles, nanomaterials, nanostructured materials, nanotechnology, nanoscience) and their explanation (Table 1). The ISO/TS 80,004 standards define nanomaterials as “materials with specific external nanoscale dimensions or with an internal nanoscale structure or surface structure”, and with a length of 1–100 nm. This definition comprises nano-objects with specific material components and nanostructured materials with an internal or surface nanoscale structure. A nanomaterial may be classified in both categories [83]. In 2011, the European Commission defined nanomaterials as natural, incidental, or manufactured materials that comprise unbound, aggregated, or agglomerated particles which higher than 50% have a size of 1–100 nm. This 50% cut-off value may be replaced by a threshold of 1–50% in particular instances justified by environmental, health, and safety concerns, or competitiveness [1,83].

## 5. Sources and Classifications of Nanomaterials

**Figure 7:**
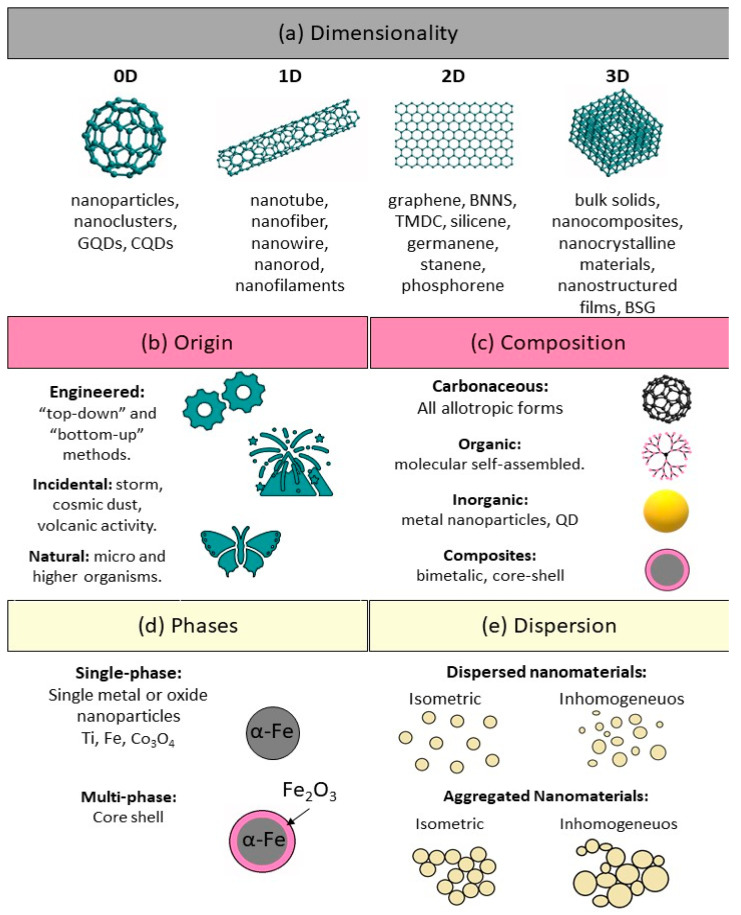
Classification of nanoparticles in function of their (**a**) dimensionality, (**b**) origin, (**c**) composition, (**d**) phases, and (**e**) dispersion. Abbreviations are Graphene quantum dots (GQDs); Carbon quantum dots (CQDs); BNNS: Boron Nitride Nano Sheets; Monolayer transition metal dichalcogenides (TMDCs); basil seed gum nanoparticles (BSG NPs); Quantum dots (QDs); Titanium (Ti); Ferric oxide (Fe_2_O_3_); Cobalt tetraoxide (Co3O4).

As there are many different sources of nanomaterials, nanomaterials must be classified and categorized nanomaterials for better understanding them. This section presents the classification of nanomaterials according to their dimensionality, origin, composition, porosity, phases, and dispersion (Figure 7).

### 5.1. Nanomaterial Classification Based on Their Dimensionality

**Figure 8:**
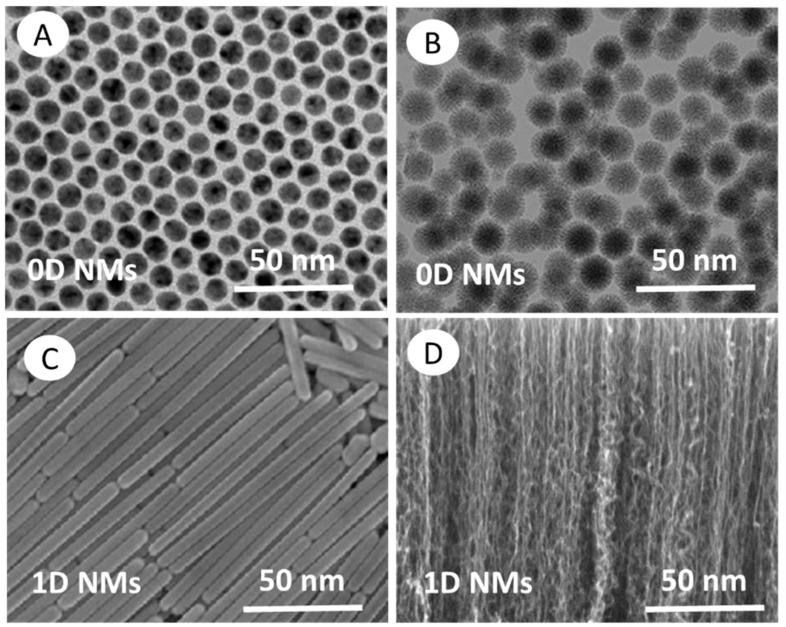

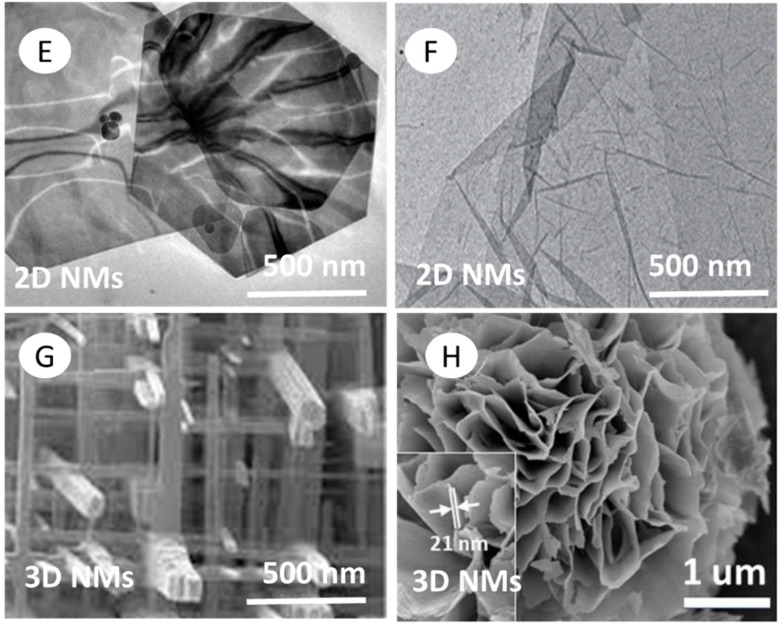
Different morphologies observed in nanomaterials with different dimensionality: (**A**) 0D nonporous monodispersed Au nanoparticles (0D) [109], © Elsevier, 2013; (**B**) 0D mesoporous silica nanoparticles (0D) [110], © MDPI, 2019; (**C**) 1D silver nanorods [111], © American Chemical Society, 2011; (**D**) 1D carbon nanotubes array [112], © IntechOpen, 2011; (**E**) 2D CuSe nanosheets [113], © Nature, 2014; (**F**) 2D graphene nanosheets [114], © Nature, 2012; (**G**) 3D WO_3_ nanowire network [115], © Beilstein Institute for the Advancement of Chemical Sciences (Germany), 2018; and (**H**) 3D flake-flower NiO nanostructures [116], © Frontiers, 2020.

Nanomaterials can be differentiated from the macroscale materials present on Earth based on their dimensionality (size and morphology) into four main types of nanomaterials (0D, 1D, 2D, and 3D) (Figure 8). Therefore, nanomaterials can be classified based on the number of dimensions that are outside the nanometer scale.

(i) *Zero-dimensional (0D) nanomaterials:* These materials have all three dimensions in the nanometer scale (i.e., <100 nm). Some examples are graphene quantum dots, carbon quantum dots, fullerenes, inorganic quantum dots, magnetic nanoparticles, noble metal nanoparticles, up conversion nanoparticles, and polymer nanoparticles. Due to their optical stability, wavelength-dependent photoluminescence, chemical inertness, cell permeability, and biocompatibility, 0D nanomaterials are interesting for optoelectronic and biomedical applications. Wang et al. (2020) [7] recently reviewed the unique properties and emerging applications of 0D nanomaterials.

(ii) *One-dimensional (1D) nanomaterials:* These materials have only one dimension >100 nm. Metal, metal oxides, and carbon-based 1D nanomaterials with a high aspect ratio (e.g., nanotubes, nanowires, and nanofibers) are excellent electron sources that emit electrons in a low electric field. Polymer nanofiber mats, veils, and webs are 1D NMs with large surface-to-volume ratio, elevated porosity and small pores that are exploited for decontamination, catalysis, filtration, and also for super-absorbent and scaffold nanomaterials for tissue engineering and wound dressings [98,99,100]. Jeevanandam et al. (2020) [101] reviewed the unique properties, emerging applications, and risks of 1D nanomaterials.

(iii) *Two-dimensional (2D) nanomaterials:* These materials type has two dimensions >100 nm. They have plate-like shapes and have thin layers with a thickness of at least one atomic layer. Graphene/graphene oxide/reduced graphene oxide, silicate clays, layered double hydroxides, transition metal dichalcogenides, transition metal oxides, black phosphorus, graphitic carbon nitride, hexagonal boron nitride, antimonite, boron nanosheets, and tin telluride nanosheets are some examples of 2D nanomaterials. Their uniform shapes, high surface-to-volume ratio (in contrast with the bulk material), and surface charge are explained by the unique physical, chemical, optical, and biological characteristics. Jin et al. (2018) [102] reviewed the unique properties, emerging applications, and risks of 2D nanomaterials.

(iv) *Three-dimensional (3D) nanomaterials:* These materials are materials with three dimensions >100 nm. This class includes but is not limited to box-shaped graphene nanostructured and bundles of nanowires, and nanotubes. In the last decade, many researchers have been focusing on the design, production, and assessment of 3D nanostructures as electrodes for electrochemical energy conversion (fuel cells) [103,104], and storage (batteries and supercapacitors) [105,106], and water treatment [107]. Materials with complex 3D structures can display unique features, such as the critical transition to bio stability of a square twist origami, and elevated mechanical strength. Complex 3D structures are important components of micro-electromechanical systems, biomedical devices, robotics, and solar cells. The synergistic integration of nanomaterials with 3D printing technologies enable the creation of architecture and devices with an unprecedented level of functional integration [108].

### 5.2. Nanomaterial Classification in the Function of Their Origin

Nanomaterials are part of the Earth system, and natural nanomaterials and living organisms have been co-evolving harmoniously. Based on their origin, Nanomaterials can be classified into four main types: “natural, incidental, bioinspired, and engineered nanomaterials” (Figure 7b). However, currently, the increasing of incidental and engineered or anthropogenic nanomaterials has altered this balance.

**Figure 9:**
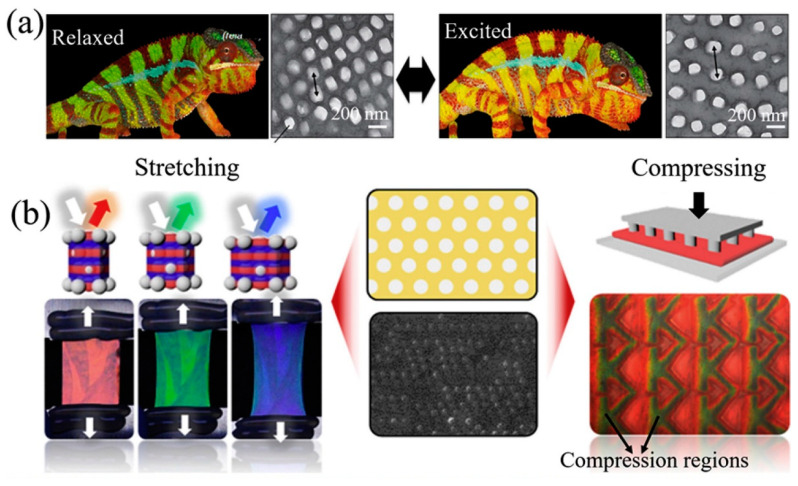
Bioinspired nanomaterials that respond to stimuli. (**a**) A chameleon in the relaxed (green color, left) and excited (yellow color, right) state. The transmission electron microscope images on the right of each panel show the periodic changes in regular arrays of guanine nanocrystals. (**b**) The color change of an artificial film upon stretching and compression [120]; © American Chemical Society, 2017.

(i) *Natural Nanomaterials*: these nanoparticles and nanostructured materials are produced by natural (bio)geochemical or mechanical processes, without any link with anthropogenic activities/processes. Some examples are the foraminifera (primarily chalk) and virus (capsid, protein) structures, the wax crystals that coat lotus or nasturtium leaves, spider and silk spider mites, the blue tarantula hues, the gecko foot spatulas, some butterfly wing scales, natural colloids (milk, blood), horny materials (claws, skin, feathers, hair), nacre, corals, and the human bone matrix. Natural inorganic nanomaterials occur by crystal growth. For example, due to their crystal structure anisotropy, clays show complex nanostructures. Moreover, volcanic activity may lead to the formation of opals, an example of naturally occurring photonic crystals due to their nanoscale structure. Natural nanomaterials sources include forest fires (combustion materials), volcanic ash, ocean spray, radon gas decline, and weathering of metal- or anion-containing rocks and acid mine drainage sites [117].

(ii) *Incidental Nanomaterials:* these nanoparticles and nanostructured materials are unintentionally produced through direct or indirect human influences or anthropogenic (e.g., mechanical or industrial) processes, such as vehicle exhaust gases, welding gases, solid fuel heating (home heathers), and combustion during cooking [118]. Incidental atmospheric nanomaterials, inadvertently formed during a deliberate procedure, might increase air pollution. Forest fires generate a wide range of nanomaterials (e.g., pigments, cement, fumed silica). It’s hard to say when human beings started making incidental nanoparticles, but probably as soon as people started taming fire. Incidental nanomaterials, byproducts of human activities, are generally have poorly controlled sizes and shapes. Incidental nanomaterials have high environmental impacts and must be considered relative to engineered nanomaterials.

(iii) *Bioinspired Nanomaterials:* these are engineered nanomaterials whose properties mimic those of natural nanomaterials or living matter. Using advanced nanofabrication technologies, many bioinspired nanomaterials with specific functions can be fabricated by modulating their structures. For example, chameleons can rapidly adjust their colors from a camouflaged state to a highly visible (excited) state (Figure 9a) when fighting or courting [119]. This color change occurs mainly by actively tuning the lattice of guanine nanocrystals within iridophore cells. Mechanochromic elastomers have been designed that mimic the photonic structure of the chameleon iridophore cells [120]. In these sensors, rigid silica nanocrystals are embedded in an elastomers matrix to form non-close-packed crystals. These sensors display a color shift from red to blue under stretching, and from red to green under compression (Figure 9b). Similar to in chameleons, this color change is reversible. These sensors could be used in wallpaper, signboards and optical recording.

(iv) *Engineered Nanomaterials:* The engineered are nanoparticles and nanostructured materials produced for specific applications based on their dimensionality and specific characteristics (e.g., nanostructured medical implants) [121,122]. These nanomaterials must be extensively characterized to reduce or minimize their unplanned negative effects [123]. The first commercial nanomaterials were engineered by aerosol (fumed silica) produced in the 1940s. In this case, the first silica nanospheres were synthesized from aqueous solutions in the 1960s. Both incidental and natural nanoparticles may have regular or irregular shapes. Usually, engineered nanoparticles have regular shapes, such as rings, spheres, tubes, etc. Engineered carbon nanostructures such as fullerenes, carbon nanotubes, and graphene in which it has a more regular shape and structure than in carbon soot (Incidental Nanomaterials).

(v) *Anthropogenic Nanomaterials:* this term refers to both incidental and engineered nanomaterials. Either the deliberate and accidental release of anthropogenic nanomaterials in the environment is becoming a major public issue.

### 5.3. Nanomaterial Classification as a Function of Their Chemical or Elemental Composition

Nanomaterials may contain one or two or more elements of the periodic table. In nature, they are usually aggregates of different elements. In function of their chemical composition, nanomaterials are differentiated into carbon, inorganic, organic, and hybrid nanomaterials. Engineered nanomaterials are typically produced using a variety of methods and may include one or more components [124].

(i) *Carbon Nanomaterials:* they are made of sp^2^-bonded carbon atoms. They include nanodiamonds, fullerenes, graphene, single- and multi-walled carbon nanotubes, carbon nanofibers, nano horns, nano-onions, and nano-graphite [125]. The synthesis methods for carbon-based materials are chemical vapor deposition (CVD), laser ablation, and arch discharge [126]. Carbon-based nanomaterials are a specific nanomaterials class because of the variety of allotropies, and they could also be considered to be organic nanomaterials because they include C-C bonds. This class also includes non-sp^2^ hybridized carbon atoms such as nano-diamonds, carbon black, and activated carbon. Most of these nanomaterials found in the environment reduce their size by milling or may be grown using a seeding growth technique (e.g., CVD for nano-diamonds) [127]. Carbon-based nanomaterials are crucial for human activities for a long time ago (e.g., combustibles, composite materials, pigments, reinforcement materials). In the sustainable energy field [128], graphite blocks are part of nuclear reactors as reflectors and moderators. Carbon nanomaterials also act as electrodes in lithium-ion rechargeable batteries and electric double-layer capacitors [129].

(ii) *Organic Nanomaterials:* they have functional properties due to the chemical association of their principal constituent (carbon) with other elements that confer specific functionalities and reactivity to the nanostructured architecture. This class includes lipid and polymer nanoparticles. Usually, both nanoparticles types are nanospheres or have a nano-encapsulated shape ranging from 10 to 1000 nm [130]. Representative examples of organic nanoparticles are dendrimers, micelles, liposomes, and ferritin. They are considered biodegradable and non-toxic nanoparticles. Lipid bilayer films are self-assemblies formed by polar lipids found in cell membranes, some microorganisms, and viruses. Langmuir–Blodgett films that mimic lipid bilayers are synthetic self-assemblies composed of amphiphilic organic molecules. In these molecules, a polar nanoblock joins with another polar block. The polar side is the head and the apolar side is the tail; both have the same dimensions [131]. These fabricated films assemble nanoparticulated micelles, liposomes, and single or bilayer films by taking advantage of the fact that the “head” is hydrophilic while the “tail” is hydrophobic. Micelles and liposomes contain a hollow core [131].

(iii) *Inorganic Nanomaterials:* They form by non-carbon elements, including metals, metal oxides, metal salts. They have different shapes (spheres, cylinders, oblates, ellipsoids, cubes, and stars) in the function of the atom packing while retaining the crystallinity nature of metal-based compounds. However, there are also amorphous inorganic nanoparticles. The surface is highly reactive and sensitive due to dangling bonds of atoms at the surface. This problem overcomes by functionalization. Among inorganic nanomaterials, metal-based quantum dots (1–10 nm) display exceptional properties due to the transition stage between bulk and few atoms [132]. Magnetic nanoparticles are also fascinating due to the high coercive fields and superparamagnetic behavior at the reduced nanoscale. Some examples of magnetic nanoparticles are magnetite (Fe_3_O_4_), γ-Fe_2_O_3_, iron (Fe), cobalt (Co), and spinel-type ferromagnets [133]. Nanoclays (2D silicates of 1 nm in thickness) are biocompatible and have low toxicity [134]. Their main application is polymer reinforcement and barriers, membrane coatings, toxin adsorption, and antibacterial and sterilizing materials [135]. Zeolite with nanosized cavities is particularly interesting for wastewater treatment where they are the charged surface leading to high ion-exchange capacity [136].

(iv) *Hybrid Nanocomposites:* they include multiphase materials with at least one nanosized component (1–100 nm), or with nanometric phase separation [137]. Hybrid nanocomposites are matrix-based, and the matrix can be a polymer, ceramic, hydrogel, or metal. At nanocomposites, the polymers are the matrix for organic or inorganic nanomaterials in different shapes. The organic or inorganic nanomaterials used as reinforcement gives specific properties to the composite. For instance, ceramic matrix composites are fiber-reinforced materials known as technical ceramics for exact applications (e.g., aerospace industry). Hydrogels display bioactive properties adaptable to precise environments, (e.g., tissue engineering), combined with the easy interaction between polymeric chains and nanostructures. Metal nanocomposites combine two or more metals. For example, intermetallic compounds, and alloys that contain nano-metals, core-shell nanoparticles, or banded components. The metal matrix may contain carbonaceous materials that apply in the aerospace industry [138,139,140].

### 5.4. Nanomaterial Classification as a Function of Their Porosity

**Figure 10:**
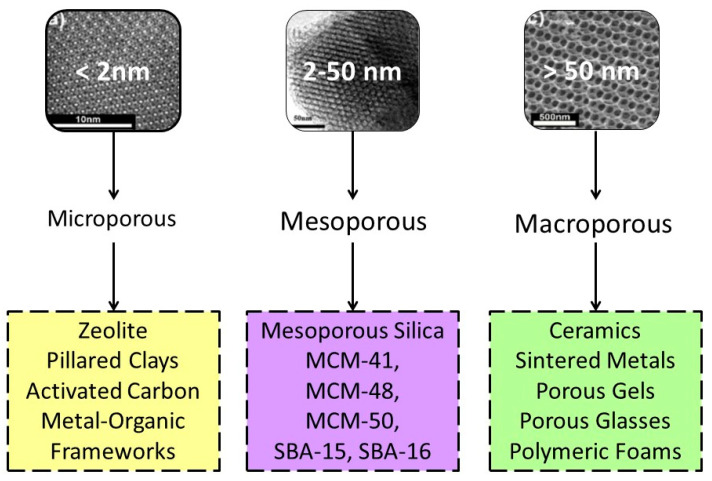
IUPAC classification of porous materials: microporous, mesoporous and macroporous structures. MCM (Mobil Composition of Matter) and SBA (Santa Barbara Amorphous).

According to IUPAC nomenclature, materials classify into three main classes based on the pore size: microporous, mesoporous, and macroporous structures (Figure 10) [141]. Nanomaterials are exploited as stationary phases for separation, resins for solid phase organic and peptide synthesis, ion-exchange resins, scavengers, enzyme supports, and catalysts [142,143].

(i) *Mesoporous materials (Super-Nanoporous):* nanoporous materials containing pores with diameters between 2 and 50 nm. According to IUPAC, a mesoporous material can have a disordered or ordered mesostructure. The porosity of mesoporous carbon is within the mesopore range, and this significantly increases the specific surface area. Activated carbon is another very common mesoporous material, typically composed of a carbon framework that can display mesoporosity and microporosity, depending on the synthesis conditions. Structures of MCM-41 (hexagonal), MCM-48 (cubic), MCM-50 (lamellar), and octomer. The SBA-15 and SBA-16 are outstanding synthetic structures of mesoporous silica. Santa Barbara Amorphous (SBA-15 and SBA-16) are a highly stable mesoporous silica sieve initially developed by researchers at the University of California at Santa Barbara. Mesoporous silica (SBA-15 and SBA-16) are promising materials for improving drug delivery due to their dual-porosity system. SBA-15 has hexagonal pores in a 2D array while SBA-16 presents a 3D body-centered cubic arrangement.

(ii) *Microporous Material:* nanoporous material with pores smaller than 2 nm in diameter. Examples of microporous materials include zeolites and metal-organic frameworks. However, it is worth to mention that micropores may be defined differently in other contexts. For example, in the context of porous aggregations such as soil, micropores are defined as cavities with a size smaller than 30 μm. Microporous silica materials with a controlled pore size and a narrow pore size distribution have been prepared by sol−gel processing using an organic-templating approach.

(iii) *Macroporous Material:* nanoporous material with pores larger than 50 nm in diameter. Hierarchical structures in which pores are of different length scales (from micro- to meso- to macro-pores) regardless of their assembly (ordered or not) are particularly interesting. However, obtaining materials with hierarchical porosity is not straightforward. Indeed, often the macroporous structure collapses during the creation of micropores [144]. The preparation of macroporous silica can be prepared using a sol–gel process but this generally requires the presence of high molecular weight, water-soluble polymers.

### 5.5. Nanomaterial Classification as a Function of Their Crystallinity

**Figure 11:**
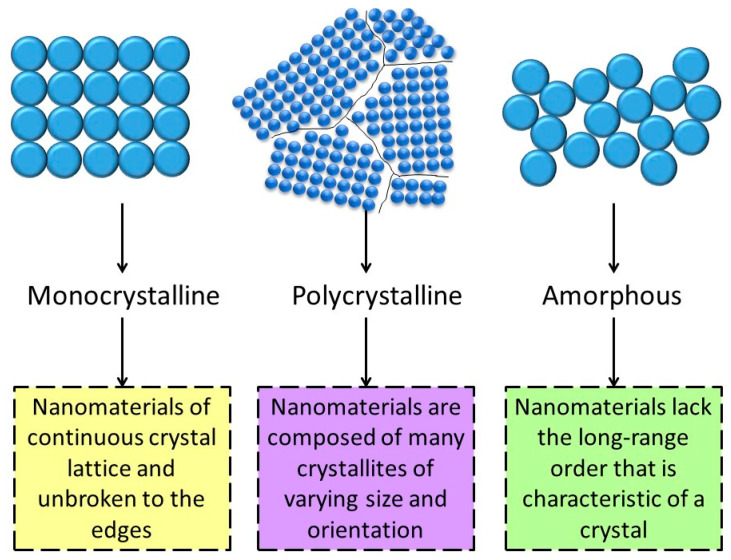
Nanomaterials Classifications based on their crystallinity.

Nanomaterials can be classified based on their crystallinity into crystalline, semi-crystalline (polycrystalline), and amorphous. In crystalline nanomaterials, the atoms in the crystal are arranged in a periodic manner. However, not all nanomaterials are nanocrystals (Figure 11). Indeed, some do not display a periodic structure (amorphous solids), and others include many small regions of single-crystal material (polycrystalline solids). In function of their crystal phases, nanomaterials can be classified in single- and multi-phase nanomaterials. Single-phase nanomaterials are nanosized materials that contain only one crystal type. Furthermore, multi-phase nanomaterials contain various crystals or semicrystalline materials. Metal nanoparticles are the best example of single-phase nanomaterials, whereas core-shell nanoparticles and nanocomposites are multi-phase nanomateirals [145,146]. Consequently, the properties of the individual phases determine the behavior of such multiphase nanomaterials.

### 5.6. Nanomaterial Classification as a Function of Their Dispersion

Nanomaterials can be classified into dispersed and aggregated nanomaterials as a function of their dispersibility and solvent type. The dispersibility of nanomaterials evaluates using the dynamic light scattering technique. The polydispersity index (PDI) describes nanomaterial particle size distribution extent. Based on the PDI value, nanomaterials can be defined as well-dispersed (PDI range: 0.1–0.3), moderately dispersed (PDI range: 0.3–0.5) and aggregated (PDI range: 0.5–1). Nanomaterials are also sub-classified in isometric (all particles have the same size) and in-homogenous (particles with different sizes) [147,148]. The dispersion stability of nanoparticles dispersions can be measured also by calculating the zeta potential. Higher zeta potential values indicate higher electrostatic repulsion between adjacent NPs in the dispersion [149]. Therefore, NP dispersions with high zeta potential (negative or positive) values are electrically stabilized, whereas NP dispersions with low zeta potential values tend to coagulate (Table 2).

## 6. Nanofabrication and Engineering of Nanomaterials

**Figure 12:**
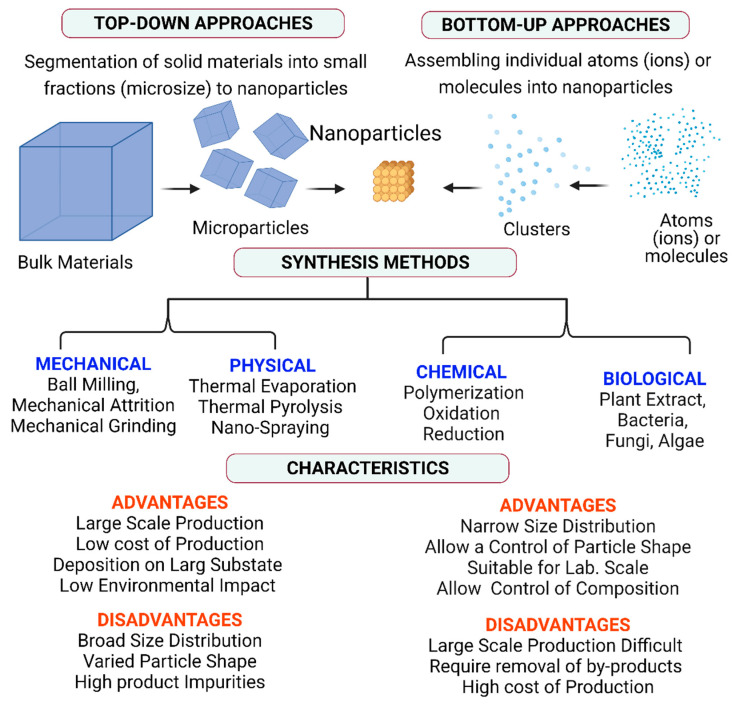
Schematic presentation showing of the different fabrication techniques to produce nanomaterials and their advances and disadvantages.

In contrast with natural nanomaterials, engineered nanomaterials are designed and fabricated in a laboratory/industry. Hence, their size, morphology, and composition are precisely controlled. Layers with a complex chemical composition also form part of this classification (e.g., a gold core covered by a silica shell, and coated by antibodies). The techniques to produce engineered nanomaterials are becoming progressively more sophisticated. However, their manufacture uses simple chemical reactions that have been known for many centuries. Indeed, their observation under microscopy revealed the deliberate and accidental production of many different nanomaterials in the past [150,151]. Many different techniques to fabricate nanomaterials have been described in the literature, with different classifications and advantages/disadvantages Figure 12.

### 6.1. Classification of Synthesis Methods Based on the Raw Materials

Nanomaterials specific features are determined by the used synthesis method. Currently, methods are grouped in “top-down” and “bottom-up” approaches, independently of the NP origin.

(i) *Bottom-up Approaches* are methods in which nanoparticles fabrication results by assembling individual atoms or molecules. These approaches exploit the atom, ion, molecule, or nanoparticles self-assembly capacity and physical and chemical interactions (e.g., hydrogen and ionic bonds, van der Waals forces, and water-mediated hydrogen bonds) to gather primary building blocks into macroscopic structures [106,152]. Wet chemical synthesis methods (e.g., sol-gel, micro-emulsion, and co-precipitation) are examples of bottom-up approaches. The main advantage of these approaches is the possibility to synthesize nanostructures with fewer defects and high homogenous chemical composition. Conversely, their major limitations are the requirement of compatible molecules and surfaces, and the lack of tools for the effective handling of molecules and atoms [36].

(ii) *Top-down Approaches*: include the segmentation of solid, large uniform materials into smaller fractions to form nanoparticles. Lithography and etching are suitable methods to produce nanostructures. Top-down approaches are suitable for synthesizing thin films and nanoparticles >100 nm in size that harbors distinct properties compared with their bulk counterparts. Top-down methods are considered superior for electronic circuitry fabrication because they allow integration and interconnection [153,154]. The resolution limit of the existing technology is still the major disadvantage of top-down approaches [155].

(iii) *Hybrid Approaches:* In these approaches, nanomaterials are produced by concomitantly employing top-down and bottom-up approaches. For example, volcanoes eject fragments of different sizes ranging from nano-fragments and nanoparticles. They are “top-down” incidental nanomaterials, and smaller fragments are precursors that might then participate in a “bottom-up” reaction to produce another material type. Lithography is a fabricating process of 1D and 2D nanostructures by replicating patterns using positive or negative masks over a substrate using photolithography, electron beam lithography, focused ion beam, soft lithography, neutral atomic beam lithography, nanoimprint lithography, and others. It is mainly considered a hybrid approach where etching is the top-down method, and ion growth layering is the bottom-up approach [156,157]. The main limitations of hybrid methods are the equipment cost and the need for highly toxic chemicals [101].

### 6.2. Classification Based on the Nature of the Deriving Forces

(i) *Mechanical Methods:* This production route includes grinding and milling. The ball milling process involves grinding macro- and micro-metric particles using high-efficiency and high-energy mills to obtain particles with non-uniform dimensions. Its major advantage is the absence of waste emission in the environment, whereas its main limitation is the lack of control over the nanoparticle size. The most common mechanical methods are (not exhaustive list): (1) monitoring and verification, in which mechanical compaction is followed by melting of the metal powder to form nanoparticles after cooling. Nanoparticles are formed by converting the bulk material into atomic structures [158]. (2) Strong deformation techniques, in which crystalline materials (e.g., metals or porcelains) are strongly deformed to enhance the nanoparticle’s hardness and ductility [159]. (3) In grinding, the starting material is subjected to extremely high energy, and ground with steel balls to obtain powdered nanoparticles. These nanoparticles have a size that varies from 3 to 25 nm [160]; (4) scrubbing in which thin silicon strips rub by immersion in chemicals (e.g., hydrofluoric acid) to obtain silicone particles on the strip surface. Then, strips are placed in a solution (e.g., isopropanol) at an ultrasound device to form nanosized droplets [159,161].

(ii) *Physical Methods:* These are “top-down” approaches used to convert bulk starting materials into smaller materials by exposure to energy (e.g., mechanical, chemical, thermal, laser irradiation). The mechanical methods be considered a subset of the physical methods. These fabrication methods include grinding, milling, physical vapor deposition, laser ablation, and spinning. Melt mixing and melt blending are mainly used for mixing two different components. To mention, polymers and nanoparticles to proper dispersion of nanoparticles in a specific matrix. In physical methods, the starting material is heated, bombarded with a beam of electrons, or thermally dissolved using laser beams. The obtained material in the vapor state is cooled using a neutral gas to increase saturation and then is immediately put on a cold surface to avoid crystal formation. Physical methods allow obtaining highly pure, smaller nanoparticles of the desired size. However, the equipment is expensive and hazardous chemicals are required. Laser ablation and spinning are commonly used physical methods [101,162]. An example of a physical method is the production of plasma using radiofrequency heating coils. It carries out placing the metal in a pestle to transfer into a vacuum chamber surrounded by radiofrequency heating coils. where it is heated above its evaporation point using helium to form plasma [163]. The metal vapor nucleates on the helium gas atoms and diffuses to a cold collector rod where nanoparticles are collected and passivated by oxygen gas [164]. In laser ablation, the starting material exposes to the extreme energy emitted by the pulsed laser. It causes the material particle volatilization and the formation of plasma that deposits on the support to form thin films [165]. Laser ablation synthesis in solution prepares colloidal solutions of nanoparticles using various solvents [166]. In the spinning method, the bulk material exposes to low pressure to deposit on a cold base. Then, a magnetic field removes smaller particles that are deposited on a support, forming a thin film [167]. Irradiation-induced synthesis of nanoparticles in solution typically performs using high-energy (1.5 MeV) electron beam irradiation. Microwave irradiation is a type of electromagnetic irradiation with mobile electric charges that is frequently employed with emulsion systems [168].

(iii) *Chemical Methods:* Chemical techniques involve the nucleation and growth of precursor species for nanomaterials fabrication. In chemical reactions in the vapor state, the material vapor enters the CVD reactor. Then, the obtained particles mix on a base at a specific temperature with other gases to form a solid strip. This method allows the preparation of mimic quasiparticles. Water and organic liquids are the most used liquid medium for these methods. Nanomaterials’ preparation results by modifying the chemical-physical balance conditions using double chemical precipitation or hydrolysis to obtain spherical nanoparticles with controlled dimensions or using sol-gel techniques and colloidal solutions at low temperatures. The dominant advantages of chemical methods are the possibility to control the nanomaterial’s size, morphology and to obtain highly stable nanomaterials. However, they require hazardous chemicals for fabrication [169,170].

(iv) *Physicochemical Methods:* These nanomaterials synthesis methods combine both physical and chemical processes. An example of a physicochemical approach is the electrochemical methods used to fabricate metal nanoparticles in which a metallic anode dissolves in an aprotic solvent. Hydrothermal, solvothermal, templating CVD, microwave irradiation, combustion, thermal decomposition, and pulsed laser deposition are physicochemical methods [171,172,173,174,175,176]. The combination of biological and chemical methods to fabricate nanoparticles also might be considered a physicochemical approach. Physicochemical processes decrease the reaction time, modulating specific size, shape, crystallinity, and stability of nanoparticles. However, they need sophisticated, high-cost equipment and hazardous chemicals [177,178].

(v) *Biological Methods:* In this method, nanomaterials fabrication develops by exploiting the activity of some microbes and plants. Biological methods are environmentally friendly approaches as these methods eliminate the use of expensive chemicals, consume less energy, and generate environmentally benign products and by products. Methods based on plants and plant extracts are more advantageous than ones using cells and microorganisms due to the low-maintenance time [179]. Although the reduction of metal ions to nanoparticles using plant extracts was already known in 1900, the production of nanometals using plant extracts and nanoparticles synthesis using living plants have been studied only in the 30 and 10 years, respectively [180]. Enzymes and other biomolecules (e.g., DNA, telomers, and actin filaments) are usually employed as catalysts for the NP growth, whereas biological organisms (e.g., fungi, bacteria, and cells) act as active units for NP production. The NP growth mechanism in these biological systems is still unknown. Nevertheless, it might be attributed to the reduction of metal salt precursors by distinct biosynthetic products, such as a-hydroxy carboxylic acids or reduced cofactors [181]. The capacity of biological synthesis to improve NP biological properties, bioavailability, bioreactivity, and biocompatibility and to reduce their toxicity is its best advantage. However, the major drawback is that the resulting NPs may lack stability, compared with those obtained using physical and chemical methods [182].

### 6.3. Classification Based on the Reaction Phase

**Figure 13:**
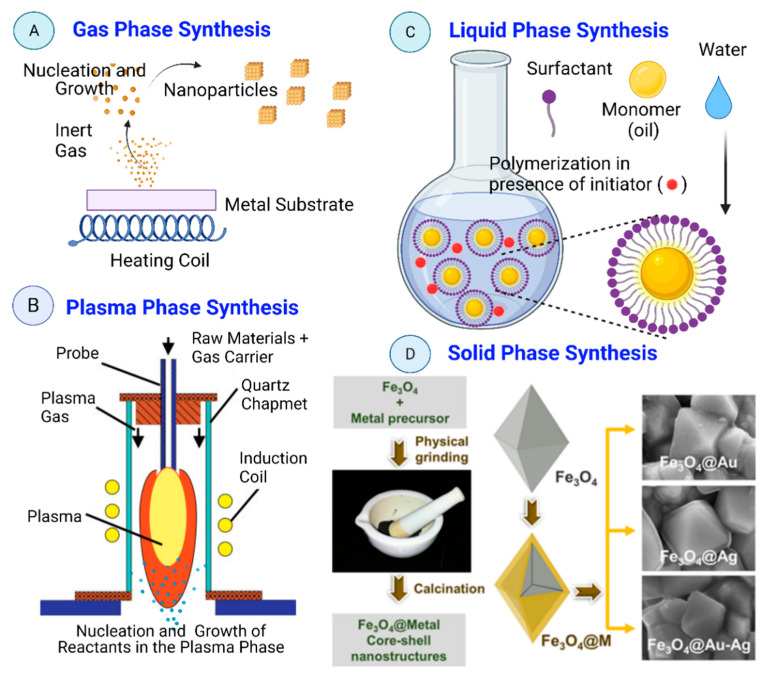
Nanomaterial production using different synthesis techniques: (**A**) Inert gas synthesis of metal nanoparticles from thermal evaporation of a metal substrate in the presence of the inert gas and then nanoparticles condensation (nucleation and growth) on a cold substrate; (**B**) Thermal plasma synthesis of NPs using different plasma-phase chemical reactions as bottom-up approaches; (**C**) Emulsion polymerization of monomers (oil phase) in water (liquid phase) in the presence of an imitator and a surfactant; (**D**) solid-phase synthesis of core-shell noble metal @Fe_3_O_4_ nanostructures [190]; © Nature, 2019.

Nanomaterial’s synthesis methods can be classified also in function of the reaction phase (gas, liquid, and solid phase) as shown in Figure 13.

(i) *Gas-Phase Synthesis:* Gas-phase synthesis of nanomaterials using bottom-up approaches (e.g., gas evaporation, exploding wire, and laser ablation process) is mainly based on chemical reactions to obtain non-volatile products that undergo homogeneous nucleation, condensation, growth, and coagulation. The main advantages of gas-phase synthesis are: (1) the possibility to obtain complex chemical structures (compared with liquid-phase synthesis); (2) particle synthesis is usually continuous (compared with batch production when using liquid and solid-phase synthesis); (3) it is cleaner than liquid-phase synthesis because solvents may contain traces of minerals or impurities. It is essential when producing nanomaterials for electronic grade semiconductors. Currently, only vacuum and gas phase synthesis approaches can avoid the presence of such impurities; (4) the possibility to modulate the nanomaterials features (e.g., porosity, size, crystallinity, stoichiometry, agglomeration, homogeneity). During gas phase synthesis of nanomaterials, the vapor mixture is thermodynamically unstable compared with the obtained nanoparticulate material in solid phase [183].

(ii) *Plasma phase synthesis*: Plasma is a charged gas made of ions, atoms or molecules with at least one orbital electron stripped (or an additional electron attached) [184]. Liquid-phase and gas-phase synthesis methods lead to the production of pollutants (e.g., waste water, carbon dioxide, nitrogen oxide, chlorine, hydrogen chloride) [2]. In plasma phase synthesis high temperatures and anhydrous conditions are used to produce particles with specific surface features and shape that cannot be obtained using other techniques. These features are required for the use of such nanomaterials in materials applications [185]. Thermal plasma physical vapor deposition might represent a cheaper nanoparticle production option compared with other gas phase techniques, but it displays important technical limitations [186] (e.g., short residence time, small high-temperature processing zone, and non-uniform high-temperature field) that need to be overcome to become a viable low-cost commercial nanoparticle production technology [187].

(iii) *Liquid-Phase Synthesis:* Nanomaterials can be produced in the liquid phase using bottom-up approaches and controlled chemical reactions. Moreover, self-limiting self-assembly processes have been developed by controlling the growth conditions [36]. In nature, nanostructures in the liquid phase can be obtained by erosion and chemical disintegration of organic or geological (clays) materials. Sol-gel processing, micro-emulsions, hydrothermal, sonochemical, chemical co-precipitation, and electrochemical methods are the most common liquid phase synthesis approaches. All need a stabilizing agent to prevent nanoparticle aggregation [188].

(v) *Supercritical-Fluid Synthesis:* A supercritical fluid is a highly-compressed fluid that combines the properties of gases and liquids. A supercritical fluid is a phase that occurs for a gas at a specific temperature and pressure such that the gas will no longer condense to a liquid. Supercritical fluid synthesis exploits unique properties of liquefied gases (solvents) in order to achieve reactions that proceed quickly to produce high-quality nanocrystals. Supercritical fluids are often referred to “green” solvents because they can proceed at moderate temperatures. these methods in flows enables continuous synthesis of metal nanomaterials and has high potential to be adapted into an industrial-level production process. Supercritical processes produce micro- or nanoparticles with narrow size distribution, and can also be used to achieve microencapsulation, surface coating of an active substance particle with a polymer or co-crystallization with excipients or host molecules. Supercritical CO_2_ and H_2_O are extensively being used in the preparation of a great variety of nanomaterials (phosphors, magnetic materials, carbon nanotubes). The greatest requirement in the application of nanomaterials is its size and morphology control, which determine the application potential of the nanoparticles, as their properties vary significantly with size.

(iv) *Solid-Phase Synthesis:* These methods are top-down approaches. They include mainly mechanical attrition and mechanochemical processes [189]. The solid-phase synthesis of nanoparticles depends on surface growth under dry or vacuum conditions. Diffusion of atoms or small clusters over suitable substrates leads to the formation of islands which are nanoparticles. Nalluri et al. synthesized core-shell Fe_3_O_4_@M (where M = Au, Ag, and Au-Ag NPs alloy) nanostructures by simple physical grinding of a metal precursor over Fe_3_O_4_ core followed by calcination, without use of solvent (Figure 13D).

## 7. Nanomaterial Features Are Influenced by Their Size and Shape

**Figure 14:**
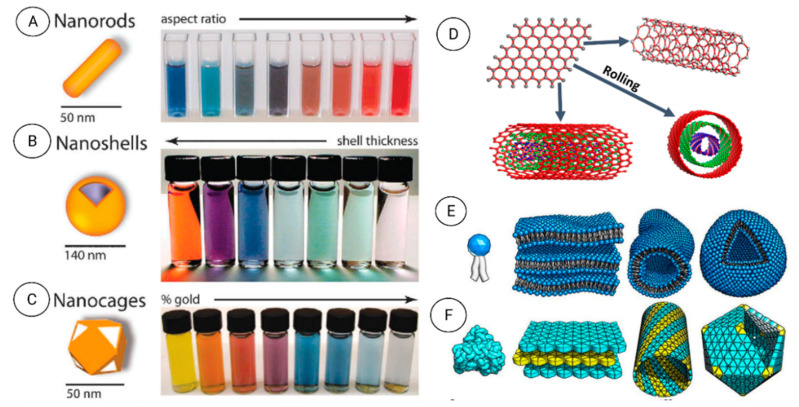
Size- and shape-dependent properties of nanomaterials: (**A**–**C**) Changes of optical properties (color) [196], © RSC, 2012.; (**D**) Rolling of graphite layer into single-walled and multi-walled carbon nanotubes [130], © Elsevier, 2019; Self-assembly of (**E**) lipids and (**F**) proteins into complex nanostructures [197], © Nature, 2017.

Individual NPs, with at least one dimension in the 1–20 nm range, usually present a single-domain crystalline lattice with very small nanocrystals, and without grain boundaries [191]. Consequently, NPs have a large volume fraction of the atoms residing in crystal faces and lower crystallinity compared with bulk materials [184]. As a result, tiny NPs (single nanocrystals) display drastically different shape- and size-dependent features (e.g., thermal decomposition, melting, electrical/thermal conductivity, magnetism, optical behavior, and catalytic properties and bioactivity) compared with the bulk material [192,193]. NPs also include a surface layer that can be functionalized by adding other small molecules (metal ions, surfactants, polymers), a shell layer that is chemically distinct from the core, and a core (i.e., the NP on its own) [194]. Therefore, much research work is axed on (i) the identification of the critical sizes below which the studied property differs from that of the bulk material and (ii) the development of simple, cost-effective, environmentally friendly, and easily scalable production methods (see Figure 14A–C) [195]. Table 3 summarizes various nanomaterials properties that can be adjusted by choosing the fabrication method.

## 8. Naturally Occurring Nanomaterials

Natural nanomaterials form in the soil, water, atmosphere, and space via physical, chemical, and biological processes. In general, natural nanomaterials are identified in the atmosphere (including the whole troposphere; nanoparticles are suspended or moved by air even at a high level), hydrosphere, lithosphere, and biosphere (micro- and macro-organisms up to humans) [208]. Anthropogenic activities lead to the release in nature of nanoparticles or precursors (in solid, liquid, and gaseous forms) that contribute to the formation of natural nanomaterials, thus altering and increasing the concentration of the nanomaterials and consequently the risks and toxicity for living organisms [209]. Table 4 lists various natural nanomaterials present in some living organisms and ecosystems.

In the Earth’s critical zone, natural nanomaterials are generated mainly by weathering and mineral formation in soils [240]. The critical zone is the outer surface of our planet that includes all terrestrial and aquatic environments, natural and modified by humans, and where complex interactions occur among living organisms, soil, water, and rocks [241]. Clays are the most abundant natural inorganic nanomaterials. Other natural nanomaterials produced by weathering (e.g., metal oxides, such iron oxides, sulfides, carbonates, phosphates) appear at a lower concentration than clays but have important biogeochemical roles [240].

**Figure 15:**
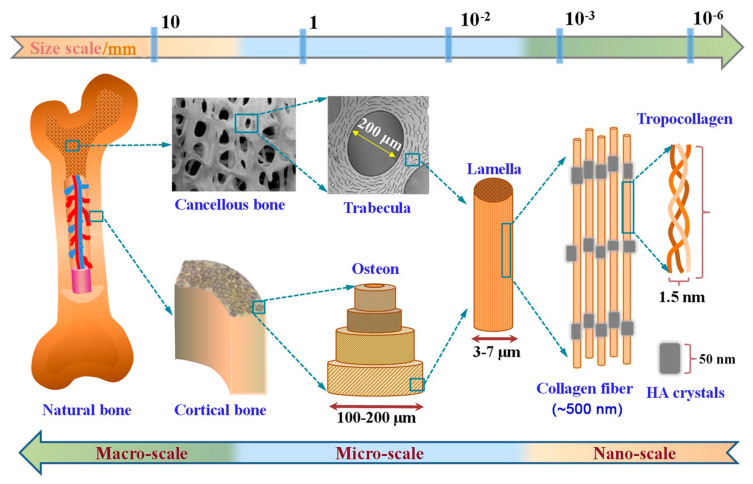
The bone hierarchical structure: cortical and cancellous bone, osteons with Haversian systems; lamellae; collagen fiber assemblies of collagen fibrils; bone mineral crystals (HA), collagen molecules, and non-collagenous proteins [248]. © Nature, 2017.

All living organisms produce/secrete specific biomolecules in the nanoscale range, such as antibodies (10–15 nm), enzymes (2–200 nm) [1,242], extracellular matrix made of Langmuir Blodget films (3–5 nm) [243]. Other molecules secreted in the body also have a nanometer size and are required for the proper functioning of the human body. Indeed, DNA and RNA (dimensions between 2.2 to 2.6 nm) are fundamental cell components needed for cell replication [1,244]. Bone is an open-cell material composed of fibrous proteins, collagen fibrils (nominally 100–200 nm) [245], calcium phosphate crystals (35 to 60 nm in diameter), and a complex vascular system. Bone organizes in a five-level structure (Figure 15) at the macro (10 mm to several cm), micro (10–500 µm), sub-nano (1–10 µm), and nano (<1 µm) scales [246,247]. Moreover, inorganic materials (mainly calcium and phosphate-containing hydroxyapatite crystals, and lower quantities of sodium, potassium, magnesium, fluorine, chlorine, and some trace elements, such as silicon, strontium, iron, zinc, and copper) serve as a support and strength to bones [248].

**Figure 16:**
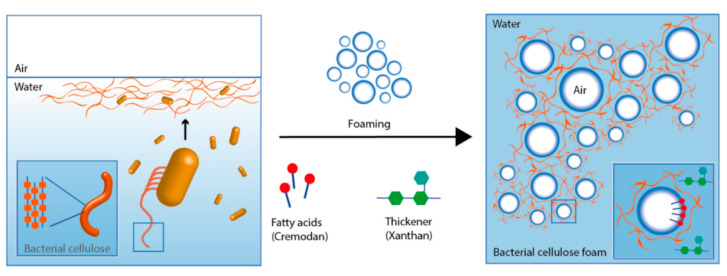
Schematic of the bacterial cellulose foam formation process. G. xylinus extrudes bacterial cellulose as a function of oxygen and migrates toward the air–water interface. To construct a bacterial cellulose foam, a suspension of G. xylinus in growth media is foamed. © Nature, 2018 [254].

Organisms with a nanoscale size (i.e., nano-organisms) are present everywhere, including the human body. The nanoscale organisms are nanobacteria, viruses (non-living organisms), fungi, algae, and yeasts that can produce nanoparticles. Viruses are among the best-studied macromolecular assemblies. Mimiviruses are among the higher viruses (capsid diameter of 400 nm) [249,250]. The length of some filoviruses, which are filamentous particles, can reach 1400 nm, with a diameter of ~80 nm. Microbial cells (bacteria, fungi, yeast), considered bioreactors, produce efficiently different nanoparticles types. They are an environmentally friendly and fast system for the synthesis of biocompatible nanomaterials [251]. Nanomaterials can be fabricated inside the cell (ions are transferred into the microbial cell to form nanoparticles in the presence of enzymes) or outside the cell (metal ions are trapped at the cell surface and reduced by enzymes) [252]. Various microorganisms tested for producing metal nanoparticles are: the bacterium Pseudomonas fluorescens (60 nm Au NPs), the yeast Yarrowia lipolytica (30 nm Au NP), the sponge Acanthella elongate (13 nm NPs), the algae Stoechospermum marginatum (50 nm NPs), and the fungus Candida albicans (20–80 NPs) [219,220,221]. As an example, Figure 16 show an efficient biosynthesis approach to produce porous structures of bacterial cellulose nanofibers (20–40 nm in diameters) by foaming a mannitol-based media with a bacterial suspension of Gluconoacetobacter xylinus [253,254].

**Figure 17:**
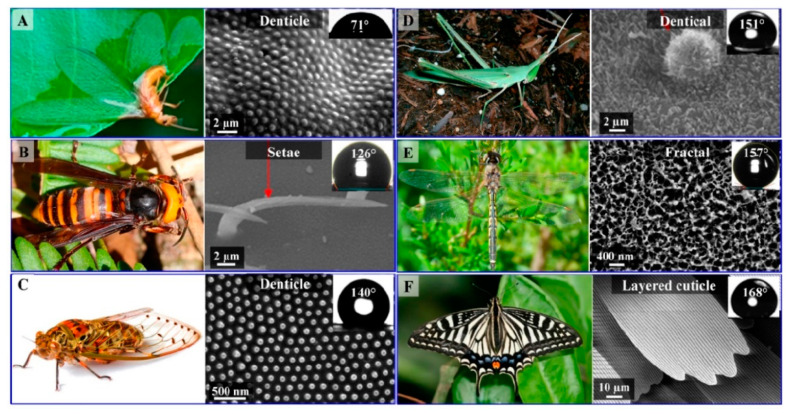
Insects and electron microscopy images (TEM and SEM) of their wings with their respective water contact angle of water droplets on the wing surface. (**A**) *Isoptera schedorhinotermes* sp.; (**B**) *Hymenoptera vespa* sp.; (**C**) *Hemiptera meimuna* microdon; (**D**) Orthoptera *Acrida* cinerea; (**E**) *Odonata hemicordulia* tau, and (**F**) Lepidoptera *Papilio Xuthus* [259]. (**B**–**E**) © Encyclopedia of Life; (**F**) © Stepanka Nemcova and Anne Ten Donkelaar. Micrographs (**B**) and (**D**) adapted with permission from [259,260]; © Elsevier, 2009. Image A and micrograph C adapted with permission from [261]; © PLoS, 2011.

Insects also contain unique nanostructures implicated in specific physical and physiological functions (Figure 17). Adhesion, chemical sensing, and response, color vision and manipulation, movement, mechano-sensation, and thermoregulation are the mentioned functions [255,256]. For instance, in fairyflies, wings have very thin hairs (300 nm–2.5 µm in diameter) that allow them to move freely at different speeds because hair spacing influences the force produced by moving. The insects wing membranes are flexible, but they are characterized by the flexural stiffness required to withstand the air mass. The wing thickness is hugely heterogeneous amongst insect’s species. For instance, it is <500 nm in fruit flies (*Drosophila* species) and >1 mm in the strong forewings of beetles (order Coleoptera) [257]. In insects, the stunning visual display is the results of the light interaction with nanostructures and is the perfect example of the nanostructure role in color vision and color manipulation (i.e., structural coloration) [256]. Photonic structures comprise at least two materials with different refractive indexes (RI) and mesoscale periodicity (i.e., 200 nm) for constructive interference of visible light [256]. In insects, these photonic structures are frequently formed by dielectric materials with low light absorption [e.g., cuticles (RI = 1.55), air (RI = 1), and pigmented layers, such as melanin layers] to obtain the appropriate RI contrast. Moreover, fireflies use bioluminescence to lure mates or for prey hunting during the night [258]. They emit a yellow to pale red light (520 to 670 nm) due to the presence of nanostructures in their body [256].

**Figure 18:**
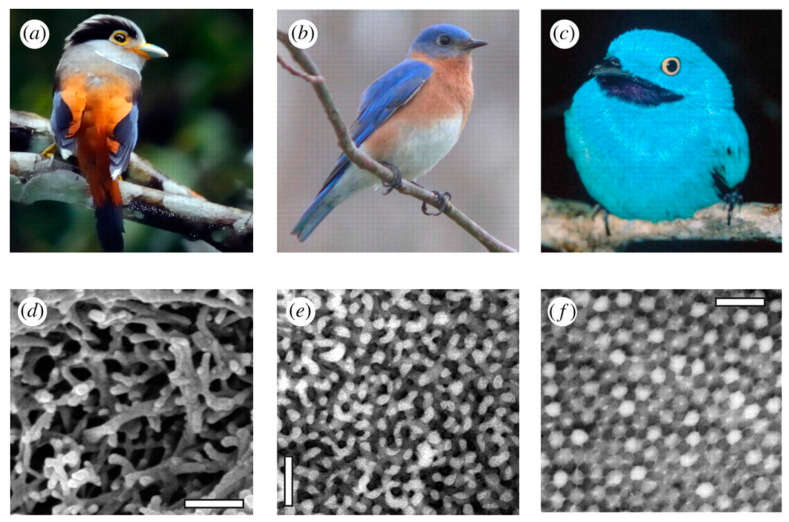
Examples of colorful feathers in birds. (**a**) Female silver-breasted broadbill (*Serilophus lunatus*, Eurylaimidae). (**b**) Male eastern bluebird (*S. sialis*, Turdidae). (**c**) Male plum-throated cotinga (*Cotinga maynana*, Cotingidae). (**d**) Scanning electron microscopy photograph of the basic nanostructure with a thin (≤1 μm) disordered layer of spongy β-keratin bars, at the periphery of medullary barb cells from the pale blue-grey primary covert feathers of *S. lunatus*, (**e**) Transmission electron microscopy photograph of the channel-type nanostructure made of β-keratin and air in the barb cells from the royal blue contour feathers of *S. sialis*. (**f**) Transmission electron microscopy photograph of the sphere-type nanostructure made of β-keratin and air in barb cells of the dark turquoise blue contour feathers of *C. maynana*. Scale bars: (**d**) 250 nm; (**e**,**f**) 500 nm. Photo credits: (**a**) Yiwen Yiwen; (**b**) Ken Thomas; and (**c**) Thomas Valqui [263]. © The Royal Society Publishing.

In birds, the complex colorful feather patches and various traits are used to attract partners as protection against predators or to recognize other birds of the same species [262]. Feather colors result from light absorption by pigments and/or a coherent light scattering of nanostructures of the feather components (keratin, melanin, and air), altering the RI of which varies periodically. Structural colors generate by the nanostructure produced by melanosomes. They are organelles with melanin that form thermodynamically stable, ordered structures. The increment of RI contrast or the relative amount of material with low RI (e.g., keratin) results in brighter or more saturated colors. For this process, the space between melanosomes increments, thus potentially reducing the order and consequently the thermodynamic stability [262]. In many birds, feathers with non-iridescent structural colors are a crucial part of their appearance (Figure 18). These colors generate by three-dimensional, amorphous (or quasi-ordered) spongy nanostructures made of β-keratin and air within the feather barb medullary cells.

**Figure 19:**
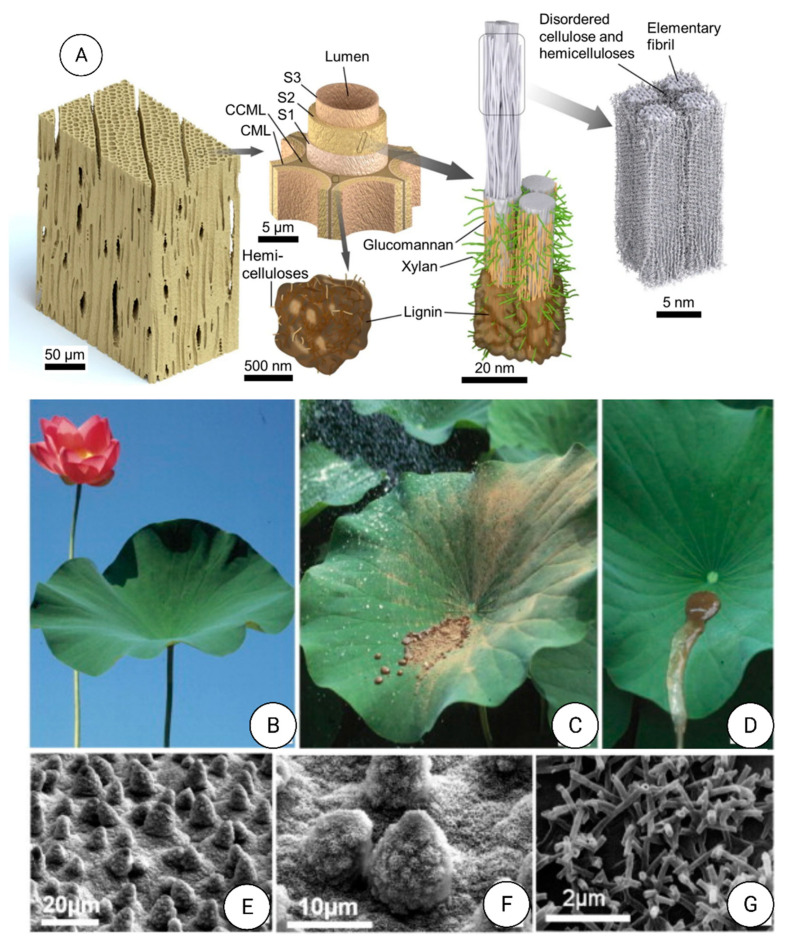
(**A**) Schematic description of softwood structure from the cellular to the nano scale. Secondary cell walls (S1, S2, and S3) are nanofiber-reinforced composites of cellulose fibrils embedded in an organized matrix of amorphous cellulose, hemicelluloses, and lignin. Cellulose is organized in highly ordered elementary fibrils (~3 nm in diameter) that are grouped into bundles to form microfibrils with amorphous polysaccharides [266]; © Nature, 2020; (**B**–**G**) Images at different magnifications of lotus leaves (Nelumbo nucifera) with self-cleaning properties [267], © Elsevier, 2009.

In plants, natural fibers are nanosized hierarchical bio-composites of cellulose fibrils present within cells. The simplest cellulose fibrils are 100–1000 nm in length and comprise crystalline and amorphous segments. Wood forms by cellulose fibrils (which give strength) embedded in a matrix of hemicelluloses and lignin. Cellulose fibrils aggregate into microfibril bundles in which the fibril crystalline and amorphous regions organize. Cellulose nanofibers (elementary fibrils and bundles) and cellulose nanocrystals (the crystalline regions) can be produced by chemical or enzymatic treatment of the plant biomass, without and with mechanical grinding, respectively [264]. Figure 19A describes the structure of softwood. The very different surface structures of flowers and grasses explained by the diverse morphology of the cells make these structures and the micro-and nanostructures present at the cell surface. For instance, lotus leaves (*Nelumbo nucifera*) have a very hydrophobic surface, water contact angle >150°) and very low water adhesion (*H* and *α*) values [265] (Figure 19B–G). Moreover, the lotus leaf self-cleaning capacity (removal of dust and particles by moving the water droplets) is explained by the Cassie-Baxter state that is linked to the leaf surface micro- and nanostructure (Figure 19E–G).

**Figure 20:**
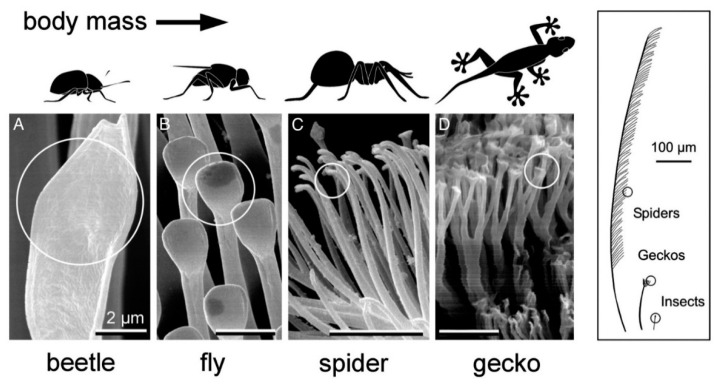
Animals and insects with widely varying body weight, such as flies, spiders, and geckos, can adhere to and move along vertical walls and even ceilings. Terminal elements (circles) in animals with hairy attachment pads. Note that adhesion structures are finer in animals with heavier body mass [268]. © PNAS, 2002.

Recently, systemic materials science studies have focused on frequent hierarchical nanostructures in animals to determine how they influence the mechanical properties of various systems (Figure 20) [268]. For instance, some lizards (geckos) and insects (beetles, flies, and spiders) can easily attach to walls and ceilings and move without falling thanks to the micro-and nano-scale structures (fibrillar adhesive system) in their feet. Geckos display the highest adhesion forces in the animal kingdom. The adhesion is due to the versatile and efficient dry adhesiveness of their feet. Electron microscopy analysis of the gecko feet shows three-level hierarchical branched nanostructures with sizes between 200 nm and 5 μm and spatula-like terminal elements [268].

## 9. Life Cycle of the Nanomaterials and Environmental Risks

**Figure 21:**
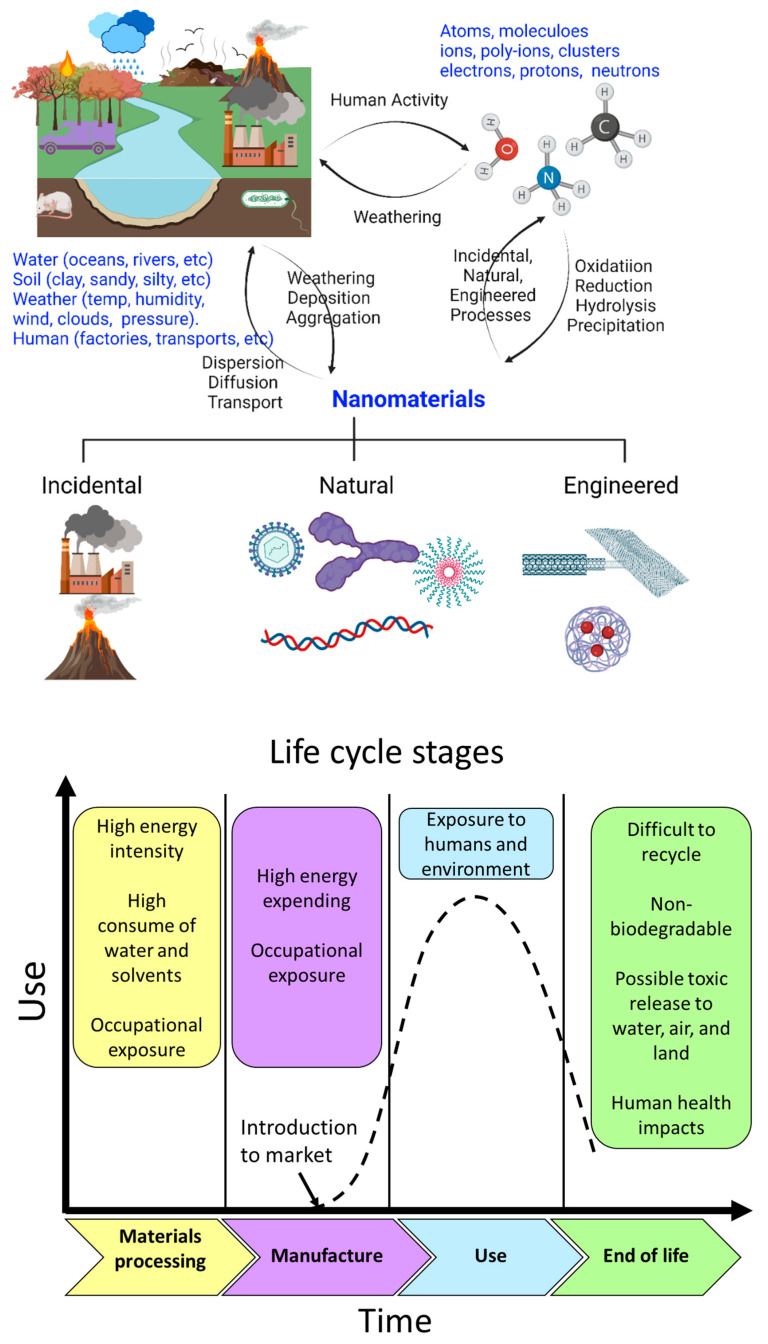
Schematic shows the life cycle of nanomaterials involve their uses over time (Material Extraction, Processing, Manufacturing, Use, and End-of-Life (Recycling/Disposal).

Nanomaterial’s production is a complex cycle during which natural, incidental, bioinspired, and engineered nanomaterials are formed. Figure 21 describes schematically the reversible cycle of the formation of natural, incidental, and engineered formation of nanomaterials. This schematic cycle includes only redox, hydrolysis, dissolution, and precipitation as the main reactions involved in nanomaterials synthesis. All these mechanisms are independent, but they are also part of a bigger system. In general, electrons transfer through a redox reaction, protons by hydrolysis, and cations and anions precipitate by dissolution and precipitation [240]. However, in the function of the external and surrounding physical-chemical conditions, other specific mechanisms also may implicated, such as gas-solid nucleation in the atmosphere, physical fragmentation, biomineralization, biochemical weathering of minerals, and photo-oxidation [269].

Nanotechnology has many advantages and benefits, but it can also present not yet fully characterized health and environmental risks due to the extensive use of nanomaterials. Therefore, life cycle-based approaches and risk assessment analyses should be combined to identify the potential problems and to adopt greener nanomanufacturing methods to protect the environment and human health (Figure 21). After use, man-made nanoparticles will end up in the environment and may cause specific adverse effects (list in Table 5). Moreover, nanoparticles exploited for environmental engineering applications (e.g., bioremediation [270], fertilizers [271], plant growth enhancers [272], seed quality improvement [273]), may also display toxicity towards the environment and the organisms present in the ecosystem [274]. In few cases, nanoparticles toxicity can be directly visualized at the environmental site. For instance, nanoparticles used for bioremediation can induce stress-related effects in plants [270]. However, some environmental risks caused by nanomaterials may not be measurable, such as the release of high ion concentration during nanomaterials degradation, or agglomeration at a later stage that may lead to indirect toxicity in the food chain and in the environment [275]. Therefore, nanomaterials long-term effects should be monitored and characterized before large-scale commercial environmental applications [276].

## 10. Toxicity of Nanomaterials

The potential effects of any nanomaterials (from natural and manufactured materials) on human health and the environment must be thoroughly characterized. In the human body, nanomaterials are recognized by the immune system that will start a biological process leading to their clearance, similar to as for any other foreign material. When these biological responses are not wanted or excessive, nanomaterials will cause toxicity. However, there are highly biocompatible nanomaterial types that can interact with the body without causing any unwanted/undesirable event (e.g., toxicity, immune reaction, thrombosis, cancer). The host response type/intensity is partly influenced by the nanomaterial’s interaction with various body components (e.g., immune cells, proteins, and lipids) and by the nanomaterial features. Therefore, a comprehensive knowledge is required to understand the relationships between the nanomaterial physical characteristics and their interactions with biological substances in order to determine its biocompatibility level.

**Figure 22:**
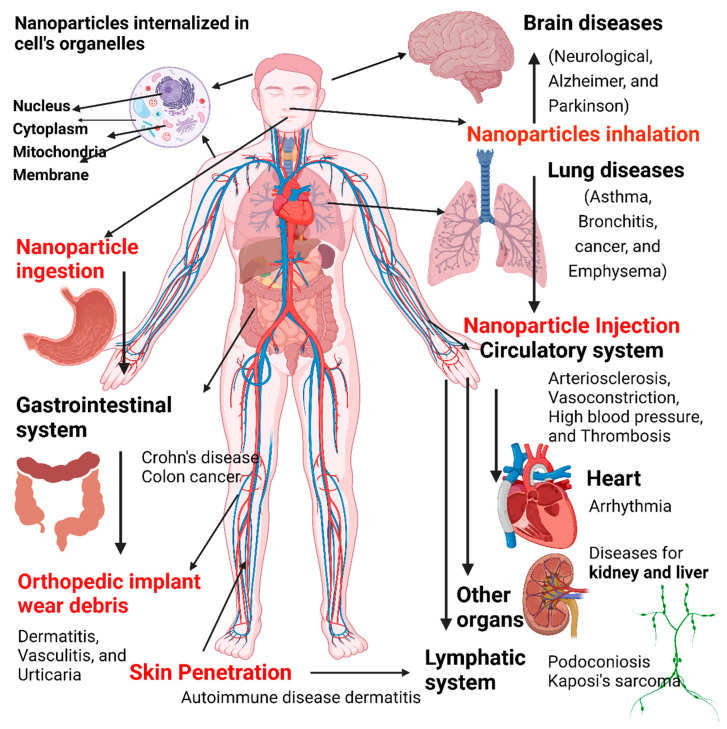
Schematic representation of the nanoparticle exposure routes in the human body, the organs/tissues concerned, and the diseases linked to such exposure (based on the findings of epidemiological, in vivo, and in vitro studies).

Nanomaterial toxicity may be influenced by the starting material composition, structure, size, shape, surface characteristics, agglomeration, purity (Table 6 and Figure 22). Therefore, it is important to investigate/predict the toxicity of any new nanomaterial and propose limitation strategies (Table 7). As different cell lines, culture conditions, and incubation times are often used to assess nanomaterial toxicity, data comparison is not straightforward, particularly to decide whether the observed cytotoxicity is physiologically relevant. Several experimental models (cultured cells, Danio rerio embryos, small mammals, such as mice and rats) are used for in vivo toxicity testing. In urban environments, particulate materials of different sizes (including in the nanoscale range) are produced by motor vehicles and other combustion sources. Much research has focused on assessing their toxic effects, leading to progressively stricter regulations. Experimental findings suggest that fine-sized particles are more harmful [191].

## 11. Nanomaterial Regulation and Challenges

As nanomaterials use is associated with the risk of unwanted events and toxicity, regulations are necessary for their manufacturing and commercial exploitation. The major challenge in NM fabrication is to use less hazardous chemicals and reduce the energy required to obtain smaller, stable, pure, and less toxic nanoparticles with better properties [1]. However, currently, no standard method is available for nanomaterials toxicological assessment, and it complicates their classification and approval for commercial applications, particularly in the biomedical sector [92]. Many countries have a nanomaterials regulatory body (Table 7) in charge of assessing the toxicity of nanomaterials because of approving them for commercial applications. However, there is no global standards organization to monitor nanomaterials toxicity, which is essential in the future to deploy some specific nanomaterial products in the global market [295,296,297,298,299].

## 12. Conclusions

Nanotechnology is a multidisciplinary discipline that involves applied physics, materials science, colloidal science, device physics, supramolecular chemistry, mechanical engineering, and electrical engineering. Nanomaterials-based tools, products, and the quality of which is certified by various regulatory bodies should offer better comfort at lower costs in the next future. Nanotechnology could be considered as the nanoscale branch of classical scientific fields, or as a newer, modern definition of existing scientific disciplines axed towards biomedical and industrial applications. Future efforts should focus on improving the features of novel nanomaterials to make them suitable for different applications. Particularly, nanotechnological research should move towards nanotechnology development in cosmetics, engineering, biomedical, and environmental industries. Moreover, scientific articles, recommendations by regulatory bodies, and patent data on nanotechnology exploitation in healthcare/personal care products should give new impetus to research. Similarly, research should focus on the development of new nanomaterials from other biopolymeric materials, nanofibrous scaffolds for effluent treatment, food industries, cosmetics, hard and soft tissue engineering applications. Currently, nanoscience and nanotechnology are rapidly developing disciplines, and their results can be exploited in many different applications, from medical/pharmacology and biotechnology to electronics/semiconductors, water purification, and university research/education. The Society of Toxicology wrote that nanotechnology discoveries are among the most important in modern natural science and they pave the way to the next revolution by developing many new materials. Moreover, nanotechnology scientists are rediscovering old materials, such as carbon, to improve consumer and industrial products, address critical energy needs, enhance security systems, and improve the medical field. The production of materials at the nanoscale leads to new, specific structural and functional features compared with the starting molecules or bulk material. This review article briefly discussed the physicochemical properties of nanomaterials. Each type of nanomaterials displays specific properties exploitable for specific applications. Therefore, a better understanding of these properties will allow the development of new and more efficient materials.

## Figures and Tables

**Table 1:** Nanotechnology definition by international organizations.

Organization	Term	Definition	Ref.
British Standards Institution	Nanoscale	Size range approximately from 1 to 100 nm.	[84]
Technical Committees of the International Standardization Organization	Nanotechnology	Scientific information used to manipulate and control nano-matter (100 nm) to exploit its size- and structure-specific features and properties that are different from those of individual atoms/molecules or bulk materials.	[85]
European Patent Office	Nanotechnology	Describes entities with a controlled geometrical size <100 nm of at least one functional component in one or more dimensions that can lead to size-dependent physical, chemical, or biological effects.	[86]
American National Standards Institute—Nanotechnology Standards Panel	Nanotechnology	Understanding and controlling matter of ~1–100 nm in size, where unique phenomena allow new applications. Nanotechnology comprises nanoscale science, engineering and technology, in which nanomaterials are imaged, measured, modeled and manipulated.	[87]
British Standards Institution	Nanoscience	Study of nano-scaled matter to understand their size- and structure-dependent features and to compare individual atoms or molecules or bulk material related differences.	[84]
European Union Scientific Committee on Consumers Products	Nanomaterials	Materials that have one or more external dimensions, or an internal structure in the nanoscale, and may display novel features compared with the same material but not in the nanoscale. Here, nanoscale means that ≥1 dimension(s) is ≤100 nm.	[88]
European Commission: Cosmetic Products Regulation	Nanomaterials	Insoluble or bio-persistent and purposely produced material with one or more external dimensions, or an internal structure in the 1–100 nm size.	[89,90]
American Chemistry Council	Engineered nanomaterial	Any purposely produced material with a size in 1, 2, or 3 dimensions between 1 and 100 nanometers. However, (i) materials without novel/unique/new features compared with the bulk materials; (ii) materials soluble in water or biologically relevant solvents at the molecular level, and (iii) micelles and single-polymer molecules are excluded.	[91]
European Commission	Nanomaterials	Any natural, incidental, or manufactured material that includes unbound, aggregated or agglomerated particles of which ≥50% are in the 1–100 nm size range. Based on specific environmental, health, safety concerns, or competitiveness issues, this threshold may be replaced by a threshold between 1 and 50%. Fullerenes, graphene flakes, and single-wall carbon nanotubes with one or more external dimensions <1 nm also are classified as nanomaterials.	[92]
European Commission for novel foods (amending Regulation No258/97), under discussion	Nanomaterials	Any purposely produced material with ≥1 dimension ≤100 nm or composed of discrete functional parts (internally or at the surface), many of which have ≥1 dimension ≤100 nm. This comprises also structures, agglomerates, and aggregates with a size >100 nm but with nanoscale-specific properties.	[92]
British Standards Institution	Nano-object	Material with one or more peripheral nanoscale dimensions.	[93]
British Standards Institution	Nanoparticle	Nano-object with three external nanoscale dimensions. The terms nanorod, nanoplate, nanosheets are used at the place of nanoparticle when the nanoobject longest and shortest axis lengths are different.	[94]
British Standards Institution	Nanofiber	Nanomaterial with two similar exterior nanoscale dimensions and a third larger dimension.	[95]
British Standards Institution	Nanocomposite	Multiphase structure with at least one phase in the nanoscale dimension.	[96]
British Standards Institution	Nanostructure	Structure of interconnected constituent parts in the nanoscale region.	[1]
British Standards Institution	Nanostructured materials	Materials with internal or surface nanostructures.	[97]

**Table 2:** Nanomaterials classification as a function of their dispersion stability in liquids.

Zeta Potential (mV)	Dispersion Stability
0–5 mV	Instable
10–30 mV	Incipient instability
30–40 mV	Moderate stability
40–60 mV	High stability
>60 mV	Excellent stability

**Table 3:** Adjustable properties of nanomaterials as a function of the synthesis method.

Properties	Example	Ref.
Catalytic activity	The catalytic activity of NPs is influenced by their size and shape. The catalyst activity is inversely proportional to the required catalytic activation energy. The catalytic activation energy of TiO_2_, SnO_2_ and CeO_2_ NPs decreases with the increase of their size. However, catalytic activation energy becomes almost size- independent when the NP size is >10–15 nm. TiO_2_, SnO_2_ and CeO_2_ NPs with a tetrahedral shape display the lowest catalytic activation energy, and therefore are more efficient catalysts.	[198]
Electrical properties	Electrical properties (e.g., conductivity or resistivity) change from the microscale to the nanoscale. The conductivity of a bulk carbon material is not influenced by its size (diameter) and twisting. Conversely, the conductivity of CNTs is modified by the cross-section area changes and upon application of shear forces (twisting). In addition, electrical conductivity of a MWCNTs is different from that of SWCNTs with the same dimensions.	[199]
Magnetic properties	The magnetic characteristics of NPs are modulated by so-called finite-size and surface effects. Finite-size effects are induced by the quantum confinement o electrons. Surface effects can be caused by the symmetry breaking of the crystal structure at the boundary of each particle, but also by the different chemical and magnetic structures of the NP core and outer shell.	[200]
Optical properties	The spectral shift of optical absorption and fluorescence properties increase the quantum efficiency of semiconductor crystals. For example, 20-nm gold (Au), platinum (Pt), silver (Ag), and palladium (Pd) NPs display a typical red wine, yellowish gray, black, and dark black color, respectively.	[201,202]
Steric features	Hollow spheres show higher selectivity for specific drug transport and controlled release. Nano drug-delivery systems (DDS) are developed to increase the drug efficiency and reduce its toxicity of loaded drugs, but only few are currently used in the clinic.	[203,204,205],
Biological features	Higher permeability through biological barriers (e.g., cell membranes, blood-brain barrier), and higher biocompatibility. NP ability to organize around proteins is mainly influenced by their size, curvature, shape and surface characteristics (charge, functionalization, and free energy).	[206,207]

**Table 4:** Natural nanomaterials in living organisms and environments.

Occurrence	Nanostructures	Thickness/Diameter (nm)	Ref.
Human body	DNA	2–2.5 nm	[210]
	Enzymes	3–7 nm	[211]
	Antibodies	10–15 nm	[212]
	Bone Collagen fibrils	60–70 nm	[213]
	Mitochondria	1000 nm	[214]
Birds	Feathers (peacock)	<100 nm	[215]
	Feathers (scarlet macaw)	<100 nm	[216]
	Feathers (toucan)	<100 nm	[217]
	Feathers (blue jay)	<100 nm	[218]
Other vertebrates and insects	Panther chameleon skin	10–100 nm	[219]
	Poison dart frog skin	10–80 nm	[220]
	Moth eyes	~100 nm	[221]
	Butterfly wings	~100 nm	[222]
	Opal weevil (beetle)	2–10 nm (Photonic Nanocrystals)	[223]
Microorganisms	Magnetotactic bacteria	Bacteria produces magnetite nanocrystals ~50–100 nm	[224]
	Bacterial Cellulose	Bacteria produces celluloses nanofibers ~20–40 nm diameter	[225,226]
	Bacteria	0.6–5000 nm	[227]
	Virus	50–150 nm	[228]
	Fungi	Setae with a spatula of 50–100 nm in size	[229]
Aquatic ecosystems	Blue-rayed limpet	Shell containing 50–100 nm particles	[230]
	Clownfish	10–100 nm	[231]
	Blue-ringed octopus	10–100 nm	[232]
	Lobster eggs	500–600 nm	[233]
Soil	Clays	3.5–5 nm	[234]
	Manganese hydrous oxide nano-minerals	3–5 nm	[235]
	Ferric iron hematite crystals	5–6 nm	[235]
	Red latosol topsoil	200 nm	[236]
Space	Lunar dust	500 nm	[237]
	Mars dust particles	50–200	[238]
	Mars soil	30–100 nm	[239]

**Table 5:** Environmental risks of nanomaterials.

Environment Type	Environmental Risks	Ref.
Air	Nanoparticles can be formed in urban areas, several combustion sources (engines, biomass burning, power generation plants) are directly emitting carbonous nanoparticles to the atmosphere. They classify as nanosized pollutants in industrialized areas.	[277]
Soil	Metal nanoparticles from the utilized biomass can be transferred to soil by nanoparticles sorption. Therefore, the high Ag concentration in the resulting biomass might restrict its agricultural use; the concentration would act as an inhibitor of bacterial growth (including the beneficial microorganisms present in the soil, such as nitrogen-fixing bacteria).	[278,279]
Water	Antimicrobial metal and metal oxide nanoparticles have a toxic effect in model organisms (e.g., nematodes, zebrafish embryos) suggesting that cumulative exposure to silver nanoparticles might significantly affect the ecological balance of aquatic environments. It is crucial to carefully assess the possible eco-toxicity when large amounts of manufactured oxide nanoparticles are released in natural waters.	[280,281,282,283,284,285]
Humans	Exposure to carbon nanomaterials and silica nanoparticles can cause lung inflammation, granuloma, and fibrosis. Carbon, Ag, and Au nanomaterials can reach also other organs (e.g., central nervous system). Quantum dots, carbon, and TiO_2_ NPs can go through the skin barrier. MnO_2_, TiO_2_, and carbon nanoparticles may enter the brain via olfactory neurons in the nasal epithelium. TiO_2_, Al_2_O_3_, carbon black, Co, and Ni NPs might be more toxic than micro-particles.	[286,287,288]
Microorganisms	Toxicities associated with nanoparticles in microorganisms are mainly related to their nanosize effect that causes membrane disorganization, generation of reactive oxygen species (ROS), and in some cases, oxidative DNA damage.	[289,290]
Plants	Nanoparticles may also adhere to the roots of the plants and cause physical or chemical toxicity to plants. Some nanoparticles were observed to be nontoxic to plant (SiO_2_ NPs), but some studies observed the toxic effect due to decrees in pH of the media after addition of these nanoparticles. The Zn- and Al-containing nanoparticles negatively affect germination and root growth of agriculturally relevant plant species.	[291,292]

**Table 6:** Factors implicated in nanomaterial toxicity.

Factor	Nanomaterial Toxicity Effect	Ref.
Dose and exposure duration	Nanoparticle’s concentration in the medium multiplied by the exposure duration directly determines the number of nanoparticles in the body cells.	[207,124]
Aggregation and concentration	Data on nanoparticles toxicity as a function of their concentration are not clear-cut. Higher nanoparticles concentrations favor their aggregation. As the size of nanoparticles aggregates is often in the micrometer range, aggregated nanoparticles may not easily enter cells, then their toxicity is decreased/abolished.	[1]
Nanoparticle size	Nanoparticles’ size influences toxicity. For instance, cell penetration and toxicity are higher for small Ag NPs (~10 nm) than for Ag^+^ ions and larger Ag NPs (20–100 nm).	[293]
Nanoparticle morphology	Nanoparticle’s toxicity varies in function of their aspect ratio. For instance, asbestos fibers of 10 µm in length may cause lung cancer, fibers between 5 and 10 µm may cause mesothelioma, and shorter fibers (2 µm) may cause asbestosis.	[294]
Surface area	Nanoparticle’s toxicity progressively increases with smaller size and larger surface area. Moreover, nanoparticles and microparticles used at the same mass dose have different effects in human cells.	[1]
Crystal structure	Nanoparticles’ crystal structure influences the cellular uptake, oxidative mechanisms, and subcellular localization. For instance, the two TiO_2_ NPs polymorphs (rutile and anatase crystal structures) display different toxic effects. In the dark, rutile TiO_2_ NPs (200 nm) cause DNA damage by oxidation and this manages not caused by anatase nanoparticles (200 nm).	[2]
Surface functionalization	Nanoparticles’ surface functionalization (hydrophilicity and surface charge) strongly influences their translocation and oxidation and DNA damage.	[266]
Pre-exposure	The phagocytic activity of cells may be promoted by shorter exposure times or by pre-exposure to NPs at lower doses (to somehow adapt the human body to that nanoparticle).	[268]

**Table 7:** Specific regulatory bodies and recommendations for assessing the nanomaterial risks.

Organization	Risks and Regulations	Ref.
Scientific Committee on Consumer Products (SCCS)—European Union	Medical standards related to ethics, environmental safety, and medical governance were revised to cover biomedical applications of nanomaterials. Currently, the most influential regulatory bodies and stricter guidelines are in the USA and EU. The European Commission (EC) published legislation and technical recommendations on nanomaterials to guarantee conformity within all EU countries and among the different sectors. As nanomaterials meet the European Registration, Evaluation, Authorization, and Restriction of Chemical (REACH) and the European Classification and Labelling of Chemicals substance definitions, their regulations also apply to nanomaterials.	[300,301]
Scientific Committee on Emerging and Newly-Identified Health Risks (SCENIHR)—European Union	The mission of SCENIHR includes assessing the risks of nanomaterials. According to the Directive 76/768/EEC of 2013, which replaced the EU cosmetics regulation 1223/2009, a nanomaterial is “an insoluble or bio-persistent and internationally manufactured material with one or more external dimensions, or an internal structure in the range of 1 to 100 nm which includes man-made fullerene, single-walled carbon nanotubes, and graphene flakes”. Moreover, several bodies in charge of cosmetics safety and regulations, such as the US Federal Food, Drug, and Cosmetic Act, Personal Care Products Council and Voluntary Cosmetic Registration Program, the EU Cosmetics Product Notification Portal, REACH and Scientific Committee on Consumer Safety, and the International Cooperation on Cosmetic Regulation highlighted the issue of the toxicity of NM contained in cosmetics.	[302,303]
Food and Drug Administration (FDA), Environmental Protection Agency (EPA) in USA	These regulatory agencies have set up protocols to monitor and manage the risks of nanomaterials and nanoproducts. Since 2006 the FDA identified nanomaterials sources, assessing the environmental impact and risks for humans, fauna, and flora, and developing protocols of nanomaterials to avoid or limit these risks.	[304]
European Medicines Agency (EMEA), and US Food and Drug Administration (FDA)	EMEA and FDA contribute to the regulation of the biomedical applications of hazardous nanomaterials. Moreover, a league of US and international advocacy groups published the book “Principles for the oversight of nanotechnologies and Nanomaterials” endorsed by 70 groups in six continents. This book advocates a thorough evaluation of all nanomaterials-derived products. It includes the need for specific NM regulations based on the precautionary principle of health and safety regulations. These principles approach for consumers and workers, transparency, public participation, and environmental protection. Likewise, the Nanomaterials Policy Recommendations report describes how to avoid or limit the risk of nanomaterials in food industries. It also suggests that industries should put in place a detailed public policy for nanomaterials use, to make public the nanomaterials safety analyses, set up supplier standards, indicate the presence of nanomaterials <500 nm in size, and implement hazard control strategies to avoid nanomaterials exposure.	[305]
UK Soil Association, Biological Farmers of Australia (BFA), Canada General Standards Board (CGSB)	These organic food/farming bodies have already forbidden the use of man-made nanomaterials in food. Scientists and industry should be aware of the existing regulation and legislation before nanomaterials production. It is acknowledged that nanomaterials are not intrinsically dangerous and that many nanomaterials are non-toxic, and others have beneficial health effects. However, future risk assessments will determine whether or not nanomaterials and derivatives are dangerous and whether additional actions are required.	[306,307]
National Nanotechnology Initiative (NNI) in USA	Nanoscale Science, Engineering, and Technology committee created the NNI to coordinate US government funding for nanotechnology research and to support the nanotechnology industry.	[1]
National Science Foundation (NSF) in USA	NSF promotes discussions on the nanotechnology risks by organizing workshops, reports, conferences, and publications.	[1]
Action Group on Erosion, Technology, and Concentration (ETC Group)—Canada	This group is at the origin of a call for a moratorium on the experimental use and commercial development of synthetic nanomaterials based on the precautionary principle, the limited knowledge on nanotechnology risks, and the absence of best practices for nanomaterials handling and use.	[1]
Chemical Industry Vision 2020 Technology Partnership	This partnership defined an R&D roadmap that focuses on (i) assessing the nanomaterials risks for human health and the environment, (ii) analyzing the risk of exposure to nanomaterials, and (iii) defining guidelines to handle nanomaterials.	[1]

## Data Availability

The data presented in this study are available on request from the corresponding author.
